# (+)-Usnic Acid and Its Derivatives as Inhibitors of a Wide Spectrum of SARS-CoV-2 Viruses

**DOI:** 10.3390/v14102154

**Published:** 2022-09-29

**Authors:** Aleksandr S. Filimonov, Olga I. Yarovaya, Anna V. Zaykovskaya, Nadezda B. Rudometova, Dmitriy N. Shcherbakov, Varvara Yu. Chirkova, Dmitry S. Baev, Sophia S. Borisevich, Olga A. Luzina, Oleg V. Pyankov, Rinat A. Maksyutov, Nariman F. Salakhutdinov

**Affiliations:** 1 Department of Medicinal Chemistry, N.N. Vorozhtsov Novosibirsk Institute of Organic Chemistry SB RAS, 630090 Novosibirsk, Russia; 2 State Research Center of Virology and Biotechnology VECTOR, Rospotrebnadzor, 630559 Yekaterinburg, Russia; 3 Department of Physical-Chemistry Biology and Biotechnology, Altay State University, 656049 Barnaul, Russia; 4 Laboratory of Chemical Physics, Ufa Institute of Chemistry Ufa Federal Research Center, 450078 Ufa, Russia

**Keywords:** usnic acid, virus SARS-CoV-2, main viral protease 3CLPro, surface glycoprotein S, molecular modeling, antiviral compound

## Abstract

In order to test the antiviral activity, a series of usnic acid derivatives were synthesized, including new, previously undescribed compounds. The activity of the derivatives against three strains of SARS-CoV-2 virus was studied. To understand the mechanism of antiviral action, the inhibitory activity of the main protease of SARS-CoV-2 virus was studied using the developed model as well as the antiviral activity against the pseudoviral system with glycoprotein S of SARS-CoV-2 virus on its surface. It was shown that usnic acid exhibits activity against three strains of SARS-CoV-2 virus: Wuhan, Delta, and Omicron. Compounds **10** and **13** also showed high activity against the three strains. The performed biological studies and molecular modeling allowed us to assume that the derivatives of usnic acid bind in the N-terminal domain of the surface glycoprotein S at the binding site of the hemoglobin decay metabolite.

## 1. Introduction

The COVID-19 pandemic, triggered in late 2019 by the SARS-CoV-2 virus and continuing around the world, has tested the community ability to respond to new challenges, both globally and locally. As of August 2022, there were more than 580 million cases and more than 6 million deaths in virtually every country in the world [1]. The impact of the pandemic on the global community is far greater than what is indicated by the reported deaths due to COVID-19 alone. Thus, of particular concern is the excess mortality of populations in many countries [2].

Until 2002, coronaviruses were considered as agents causing non-serious upper respiratory tract diseases (with rare fatalities). The new coronavirus SARS-CoV-2 is presumably a recombinant virus between a bat coronavirus and a coronavirus of unknown origin. The pathogenesis of COVID-19 disease caused by SARS-CoV-2 has been described in detail in numerous review papers [3]. There are several strategies for combating human viral diseases. The first is disease prevention, which includes vaccination and a set of sanitary and epidemiological measures. An alternative to prophylactic vaccination can be the use of specific antiviral drugs.

Theoretically, each stage of the virus life cycle can be a potential target for drug therapy. The current search for new agents with specific antiviral activity is focused on two main directions. The first is the search for inhibitors of virus entry; the second is the search for inhibitors of intracellular replication [4]. To study entry inhibitors, pseudoviral systems have recently been widely used in modern medicinal chemistry [5]. A pseudovirus is a recombinant particle consisting of the capsid of one virus (usually lentivirus or vesicular stomatitis virus) with the proteins of another virus on its surface. Binding and penetration of such a pseudoviral particle into the cell is fully provided by the surface protein. Such particles are obtained artificially, by introducing various gene-engineered constructs into special progenitor cells. Pseudotyping is achieved using plasmids encoding various surface proteins [6]. The use of pseudoviral systems is especially important in the search for specific agents active against particularly dangerous viruses, such as Ebola, Marburg, or SARS-CoV-2 coronovirus [7].

The SARS-CoV-2 virus genome encodes more than twenty proteins, among which there are two proteases-papain-like PLpro and 3-chymotrypsin-like 3CLpro, which are also called the main proteases [8]. These proteases are vital for viral replication and their function is to cleave two translatable viral polyproteins (1A and 1AB) into functional components. Because of their small size, as well as their high homology to similar proteins of the coronaviruses that cause SARS and MERS atypical pneumonias, the major protease of SARS-CoV-2 is the most characterized target for potential antiviral drugs [9]. The main protease cleaves polyprotein 1AB at 11 specific sites. The site recognition sequence in most cases consists of the (Leu-Gln)-(Ser-Ala-Gly) chain site, where the bond between glutamine and serine is cleaved. Inhibition of the activity of the main protease leads to a halt in viral replication. No proteases with the same cleavage specificity are known among human enzymes, which may indicate the probable lack of toxicity of potent inhibitors of the main protease of SARS-CoV-2 [10].

Current trends in antiviral drug development usually focus on repurposing drugs against potential virus targets. The use of natural compounds as starting platforms in the synthesis of new compounds with antiviral properties is a promising direction in medicinal chemistry [11]. In the last two years, special attention has been focused on the search for inhibitors of the SARS-CoV-2 virus among natural compounds and their derivatives [12,13]. The development of new effective chemotherapeutic agents aimed at inhibiting specific enzymes critical to the viral life cycle is essential for the successful etiotropic treatment of patients with infectious diseases, most of whom receive only symptomatic and supportive therapies. Key enzymes of the viral life cycle often have a very conservative structure within the whole family, which provides additional advantages in the development of drugs with a broad spectrum of action.

**Figure 1:**
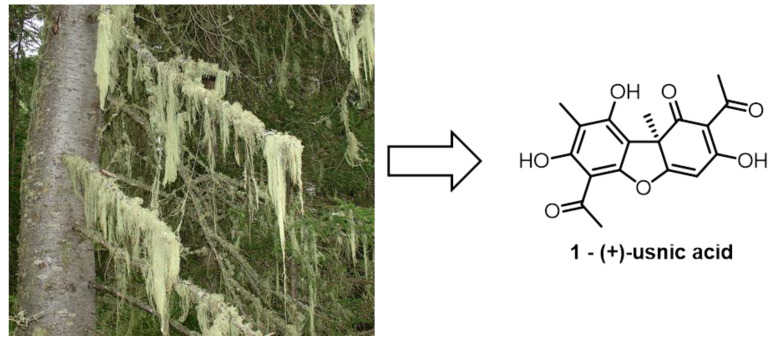
**Left**: *Usnea longissima*; **Right**: structure of (+)-usnic acid **1**.

In this work, (+)-usnic acid was used as a starting compound, Figure 1.

Usnic acid is a secondary metabolite of the lichen species *Usnea*, *Cladonia*, *Alectoria*, and many others. It has a wide range of biological activities: antimicrobial, antitumor, anti-inflammatory, and antiviral [14]. Earlier it was shown that (+)-usnic acid shows activity against Epstein–Barr virus and rat polyomavirus [15]. In a series of studies it was found that (+)-usnic acid exhibits activity against the H1N1 influenza virus, and its chemical modification can lead to substances with more pronounced anti-influenza properties [16]. Thus, the results of [17,18] show that the antiviral activity of some (+)-usnic acid derivatives is confirmed in in vitro and in vivo tests. It was shown that administration of one of the derivatives is capable of reducing the lethality among infected mice, and also does not lead to the emergence of resistant strains.

Recent in silico studies demonstrate the ability of (+)-usnic acid to bind to the active site of the 3CLpro protease [19], as well as to the receptor-binding domain (RBD) of the SARS-CoV-2 virus surface glycoprotein S [20]. In another work, it was shown that usnic acid can bind to transmembrane serine protease 2 (TMPRSS2) [21]. Recently, the activity of (+)-usnic acid and its salts against the SARS-CoV-2 virus was studied using immunofluorescence analysis [22]. The above data indicate the potential for the study of usnic acid and its derivatives as antiviral agents. In the present work, we synthesized a set of both the previously described and new (+)-usnic acid derivatives to study their activity against the SARS-CoV-2 coronavirus.

## 2. Materials and Methods

### 2.1. Chemistry

The analytical and spectral studies were performed at the Chemical Service Center for the collective use of Siberian Branch of the Russian Academy of Science.

The ^1^H and ^13^C-NMR spectra for solutions of the compounds in CDCl_3_ were recorded on a Bruker AV-400 spectrometer (400.13 and 100.61 MHz, respectively). The residual signals of the solvent were used as references (δ_H_ 7.24, δ_C_ 76.90 for CDCl_3_). The mass spectra (70 eV) were recorded on a DFS Thermo Scientific high-resolution mass spectrometer. The melting points were measured using a Kofler heating stage. The specific rotation was determined on a PolAAr 3005 (Optical Activity Ltd., Huntingdon, UK) and provided in (deg × mL) × (g × dm)^−1^, whereas the concentration of solutions is shown in g × (100 × mL)^−1^. Merck silica gel (63–200 μ) was used for the column chromatography. The thin-layer chromatography was performed on TLC Silica gel 60F_254_ (Merck KGaA, Darmstadt, Germany).

(*R*)-(+)-Usnic acid **1** was obtained from the Zhejiang Yixin Pharmaceutical Co., Ltd. company, China. Synthetic starting materials, reagents, and solvents were purchased from Sigma Aldrich, Acros Organics, and AlfaAesar (95–99% pure). All chemicals were used as described unless otherwise noted. Reagent-grade solvents were redistilled prior to use.

Derivatives with the isoxazole cycle **2** and **3** were synthesized according to [23]. Bromousnic acid **4** was synthesized according to [24]. Thioether **5a** was synthesized according to [25]. Derivative **6** was synthesized according to [26]. Amino derivative of usnic acid **7** was synthesized according to [27]. Furanon **8** was synthesized according to [28].

#### 2.1.1. Procedure for the Synthesis of Compound **5b**

Potassium hydroxide (1.1 mmol) and corresponding thiol (1.1 mmol) were dissolved in methanol (6 mL). The resulting mixture was stirred at room temperature for 10–15 min. Then, the resulting solution was added to the solution of compound **4** (1 mmol) in 2 mL of methylene chloride and stirred at room temperature for 2–3 h. The course of reaction was controlled by TLC. The reaction mixture was washed 2 times with distilled water, dried over MgSO_4_, and concentrated. The resulting residue was separated on silica gel (eluent: methylene chloride).The product was separated as a diastereomere mixture. 

(2S)-2-({2-[(1R)-12-acetyl-3,5,11-trihydroxy-1,4-dimethyl-13-oxo-8-oxatricyclo[7.4.0.0^2,7^]trideca-2(7),3,5,9,11-pentaen-6-yl]-2-oxoethyl}sulfanyl)propanoic acid and (2R)-2-({2-[(1R)-12-acetyl-3,5,11-trihydroxy-1,4-dimethyl-13-oxo-8-oxatricyclo [7.4.0.0^2,7^]trideca-2(7),3,5,9,11-pentaen-6-yl]-2-oxoethyl}sulfanyl)propanoic acid (1:1) (**5b**): Yellow amorphous powder. Yield: 72%. M.p. 91–95 °C. δ_H_ (CDCl_3_, J Hz): 1.45 (3H, t, J = 7.35), 1.73 (3H, s), 2.07 (3H, s), 2.63 (3H, s), 3.57 (1H, m), 3.98–4.14 (2H, m), 5.95 and 5.96 (1H, s), 10.19 (1H, ss), 11.11 (1H, s), 12.85 and 12.86 (1H, s), 18.80 (1H, s). δc (CDCl_3_): 7.47, 16.44 and 16.52, 27.79, 31.88 and 31.94, 40.55 and 40.70, 40.97, 58.75 and 58.77, 98.51, 99.70 and 99.75, 104.17, 105.05 and 105.07, 109.5, 154.45, 157.89, 164.09, 178.54 and 178.63, 178.60, 191.49, 196.23 and 196.28, 197.75 and 197.77, 201.69. HRMS: m/z: [M]^+^ calcd for C_21_H_20_O_9_^32^S_1_ 448.0823. Found: M = 448.0827. α_D_^23^ +314 (c 0.108, CHCl_3_).

#### 2.1.2. Procedure for Synthesis of Compound **10**

Potassium hydroxide solution (50%, 0.5 mL) was added dropwise to the solution of compound **9** (1 mmol) in methanol (10 mL) while stirring in an ice bath (−4 °C). After 1 h mixture was diluted with water and hydrochloride solution (1 M) to a pH of 4. The precipitate was filtered of, washed with water, and air dried. The product was isolated after column chromatography on silica gel (eluent: dichloromethane).

(2R)-4-acetyl-5,11,13-trihydroxy-10-(2-hydroxyacetyl)-2,12-dimethyl-8-oxatricyclo[7.4.0.0^2,7^]trideca-1(9),4,6,10,12-pentaen-3-one (**10**): Yellow amorphous powder. Yield: 62%. M.p. 107–110 °C. δ_H_ (CDCl_3_, J Hz): 1.73 (3H, s), 2.09 (3H, s), 2.64 (3H, s), 4.78 (2H, s), 5.98 (1H, s), 11.11 (1H, s), 12.45 (1H, s), 18.83 (1H, s). δc (CDCl_3_): 7.44, 27.78, 29.58, 31.90, 58.71, 67.52, 98.27, 98.84, 104.29, 105.09, 105.12, 109.51, 154.93, 158.28, 163.09, 178.62, 191.53, 197.71, 199.40, 201.72. HRMS: m/z: [M]^+^ calcd for C_18_H_16_O_8_ 360,0844. Found: M = 360,0840. α_D_^23^ +378 (c 0.108, CHCl_3_).

#### 2.1.3. Procedure for Hydrogenation of Usnic Acid

Usnic acid **1** (2 g) was added to 25 mL of THF. After the substance dissolved, a catalyst (Pd/C 10%) was added to the mixture. A three-way crane was placed on the flask. One output is connected to hydrogen, another is connected to a vacuum pump. The air from the flask was removed by vacuum. Then, the system was filled with hydrogen and was stirred for 5 min. The procedure was repeated once. The obtained mixture was stirred in the hydrogen atmosphere overnight. After that, the mixture was filtered out, and the solvent was removed. The products were isolated after column chromatography.

(2R,7R)-4,10-diacetyl-5,11,13-trihydroxy-2,12-dimethyl-8-oxatricyclo [7.4.0.0^2,7^]trideca-1(9),4,10,12-tetraen-3-one (**11**): Yellow amorphous powder. Yield: 35%. The spectrum of the substance corresponds to the literature [29].

(2R,7R)-10-acetyl-4-ethyl-5,11,13-trihydroxy-2,12-dimethyl-8-oxatricyclo[7.4.0.0^2,7^]trideca-1(13),4,9,11-tetraen-3-one (**12**): Yellow amorphous powder. M.p. 58–62 °C. Yield: 36%. δ_H_ (CDCl_3_, J Hz): 0.95 (3H, t, J = 7.5), 1.59 (3H, s), 2.01 (3H, s), 2.27 (1H, dq, J_1_ = 7.5, J_2_ = 7.0), 2.33 (1H, dq, J_1_ = 7.5, J_2_ = 7.0), 2.55 (3H, s), 2.89 (1H, dd, J_1_ = 6.0, J_2_ = 17.6) and 2.99 (1H, dd, J_1_ = 6.0, J_2_ = 17.6) (AB-system), 4.83 (1H, dd, J_1_ = 6.0, J_2_ = 6.0), 9.63 (1H, ss), 13.35 (1H, s). δc (CDCl_3_): 7.14, 12.77, 15.44, 23.89, 31.11, 31.90, 51.77, 84.75, 101.67, 105.84, 106.05, 116.92, 159.19, 159.70, 162.95, 170.0, 198.3, 201.89. HRMS: m/z: [M]^+^ calcd for C_18_H_20_O_6_ 332.1254. Found: M = 332.1249. α_D_^23^ -21 (c 0.096, CHCl_3_).

(2R,4E,7R)-10-acetyl-11,13-dihydroxy-4-(1-hydroxyethylidene)-2,12-dimethyl-8-oxatricyclo [7.4.0.0^2,7^] trideca-1(13),9,11-trien-3-one (**13**): Yellow amorphous powder. Yield: 15%. M.p. 100–103 °C. δ_H_ (CDCl_3_, J Hz): 1.59 (3H, s), 1.99 (3H, s), 1.99–2.07 (2H, m), 2.28–2.40 (1H, m), 2.34 (1H, ddd, J_1_ = 4.8, J_2_ = 7.0, J_3_ = 15.0) and 2.48 (1H, ddd, J_1_ = 4.8, J_2_ = 7.0, J_3_ = 15.0) (AB-system), 2.55 (3H, s), 4.69 (1H, dd, J_1_ = 4.6, J_2_ = 6.9), 9.38 (1H, s), 13.42 (1H, s), 16.47 (1H, s). δc (CDCl_3_): 8.05, 20.2, 22.90, 25.20, 27.05, 32.24, 52.67, 89.89, 102.62, 106.42, 106.64, 106.70, 160.02, 160.07, 164.04, 191.74, 195.06, 202.47. HRMS: m/z: [M]^+^ calcd for C_18_H_20_O_6_ 332.1254. Found: M = 332.1253. α_D_^23^ -262 (c 0.156, CHCl_3_).

#### 2.1.4. Procedure for the Synthesis of Compound **14**

Dihydrousnic acid **11** (1.0 mmol) and hydroxylammonium chloride (1.1 mmol) were dissolved in pyridine (1 mL). The resulting mixture was stirred under reflux for 1.5 h. After that, the reaction mixture was cooled, and aqueous hydrochloric acid was added to the mixture. The precipitate was filtered off, washed with water, and then air dried. The resulting solid was purified by chromatography over silica gel with CH_2_Cl_2_.

(1R,9R)-6-acetyl-3,5-dihydroxy-1,4,14-trimethyl-8,12-dioxa-13-azatetracyclo[7.7.0.0^2,7^.0^11^,^1^⁵]hexadeca-2(7),3,5,11(15),13-pentaen-16-one (**14**): Yellow amorphous powder. Yield: 54%. M.p. 152–156 °C. δ_H_ (CDCl_3_, J Hz): 1.73 (3H, s), 2.03 (3H, s), 2.46 (3H, s) 2.52 (3H, s), 3.43–3.64 (2H, m), 5.10 (1H, m), 8.88 (1H, s), 13.42 (1H, s). δc (CDCl_3_): 6.84, 10.24, 21.67, 25.26, 30.93, 54.53, 86.68, 101.48, 105.22, 17.10, 113.08, 157.65, 158.36, 158.50, 163.15, 177.68, 194.64, 201.03. HRMS: m/z: [M]^+^ calcd for C_18_H_17_O_6_N_1_ 343.1050. Found: M = 343.1053. α_D_^23^ +113 (c 0.156, CHCl_3_).

### 2.2. Biological Experiments

#### 2.2.1. Evaluation of the Antiviral Activities against SARS-CoV-2 Viruses

Studies using the SARS-CoV-2 virus have been performed in laboratories with BSL-3 containment. The study was carried out using following three coronavirus strains: SARS-CoV-2 nCoV/Victoria/1/2020 (GISAID ID: EPI_ISL_406844, lineages B), hCoV-19/Russia/PSK-2804/2021 (GISAID ID: EPI_ISL_7338814, lineages B1.617.2), and hCoV-19/Russia/Moscow171619-031221/2021 (EPI_ISL_8920444, lineages B.1.1.529) (State collection of pathogens of viral infections and rickettsioses of SRC VB “Vector” Rospotrebnadzor, RF).

The viruses were grown in a Vero E6 cell culture. The infectivity titer of the virus stock was measured at 7.0, 6.5 at 5.5 lgTCD_50_/mL for strains nCoV/Victoria/1/2020, hCoV-19/Russia/PSK-2804/2021 at hCoV-19/Russia/Moscow171619-031221/2021, respectively. Vero E6 cells were grown in 96-well culture plates to a confluence of at least 95%. Samples were dissolved in dimethyl sulfoxide (DMSO) to a concentration of 10 mg/mL. Remdesevir was used as a control drug. The effective (IC_50_) concentrations of compounds were evaluated in the test to reduce the cytopathic effect on cells. Serial three-fold dilutions of the compounds were prepared, starting at a concentration of 600 μg/mL. To test each compound, the virus doses of 100 TCD_50_ per well were used.

Inhibitory activities and toxicities of the tested compounds were assessed simultaneously. Specifically, dilutions of the compounds were added to the wells in the culture plates containing a monolayer of cells. A plain medium (to determine the toxic concentration of the tested compounds) or a medium containing a virus (to determine inhibitory activities) was then added. The culture plates were incubated at 37 °C for 4 days; after which they were stained using the MTT assay protocol. The results were recorded with ThermoScientificMultiskanFC; data processing was carried out in the SOFTmax PRO 4.0 program using a 4-parameter analysis method. The 50% toxic concentration (CD_50_) and the 50% inhibition (IC_50_) concentration were both determined.

#### 2.2.2. Evaluation of Inhibitory Activity against the Main Viral Protease

To assess the ability to inhibit the main protease (3CLpro), the IC_50_ is the semi-inhibitory concentration of the substance, at which the fluorescence level is reduced by 50% relative to the value obtained without adding the inhibitor [30]. Fluorescence occurs due to cleavage of the peptide substrate DabcylKTSAVLQ↓SGFRKME(Edans)NH2 by 3CLpro protease. In the study, the signal was recorded using the CLARIOstar Plus instrument (BMG Labtech) at 355 and 460 nm for excitation/radiation, respectively, in kinetic scan mode. Reaction mixtures containing TrisHCl buffer, fluorogenic substrate, 3CLpro, and the compound being tested were prepared and incubated for 30 min in a 384-well plate at 30 °C. The instrument was calibrated using a solution of the peptide that had undergone complete hydrolysis. The accompanying MARS Data Analysis software (https://www.selectscience.net/products/mars-data-analysis-software/?prodID=81306, accessed on 18 August 2022) was used to calculate IC_50_.

#### 2.2.3. Evaluation of Antiviral Activity Using the Pseudovirus System

##### Cell Cultures

The HEK293T cell line was provided by the Department “Collections of Microorganisms” of the Rospotrebnadzor State Research Center Vector (Koltsovo, Russia). The HEK293-hACE2 cell line was provided by the Institute of Molecular and Cellular Biology SB RAS (Novosibirsk, Russia). The HEK293T-hACE2-TMPRSS2 (transient) was obtained by transfecting 293 cells with the pDUO-hACE2-TMPRSS2 plasmid. Cells were cultured on Dulbecco’s Modified Eagle Medium (DMEM) (Invitrogen) and Dulbecco’s Modified Eagle Medium/Nutrient Mixture F-12 (DMEM/F12) (SRC Vector, Russia), with the addition of 10% (*v/v*) thermally inactivated fetal cow serum (Invitrogen, Carlsbad, CA, USA), and 0.6 mg/mL L-glutamine (Invitrogen, Carlsbad, CA, USA) and 50 µg/mL gentamicin.

Vero E6 cells were used in the experiment (cells of the renal epithelium of an African green monkey) (collection of SRC VB “Vector” Rospotrebnadzor, RF). The cells were cultured in a DMEM medium (Gibco) with L-glutamine, with 10% fetal calf serum (Gibco) and antibiotic-antimycotic (Gibco) at 37 °C, 5% CO_2_.

##### Plasmids

A second-generation lentiviral system was used to generate pseudoviruses. The psPAX2, which provides formation of lentiviral particles (Addgene #12260), was used as a packaging plasmid. The Ph-SΔ18 encoding the SARS-CoV-2 protein was used as the envelope plasmid, and was obtained by inserting the nucleotide sequence encoding the S protein of SARS-CoV-2 (GenBank:MN908947) into the phMGFP vector. The last 18 amino acids of the S protein sequence were deleted, and then the codon composition was optimized using the GeneOptimizer tool (https://www.thermofisher.com/ru/en/home/life-science/cloning/gene-synthesis/geneart-gene-synthesis/geneoptimizer.html (accessed on 20 February 2020)). The final nucleotide sequence was synthesized by DNA-Synthesis LLC. The insertion was performed at the NheI and AsiGI sites. In addition, a D614G mutation was introduced into the amino acid sequence of the S protein. The reporter plasmid pLenti-Luc-GFP was obtained from the lentiviral vector pCDH-EF1a-GaussiaSP-MCS-IRES-copGFP (kindly provided by T.N. Belovezhets (ICBFM SB RAS)) by replacing the Gaussia luciferase sequence with that of firefly luciferase. For this purpose, PCR amplification of the firefly luciferase nucleotide sequence was performed using the primers Lenti-Luc-F 5′-aaaaaatctagctagccaccatggaagatgcca-3′ and Lenti-Luc-R 5′-aaaaaaggatccttacacggcgatcttgccg-3′. Plasmid pCAG-luciferase (Addgene #55764) was used as a matrix. Next, the PCR product was inserted into the pCDH-EF1a-GaussiaSP-MCS-IRES-copGFP plasmid at the XbaI and BamHI restriction sites. The pDUO-hACE2-TMPRSS2 plasmid was purchased from the commercial firm Invivogen (San Diego, CA, USA).

##### Preparation of SARS-CoV-2 Pseudotyped Lentiviral Particles

To obtain SARS-CoV-2 pseudotyped lentiviral particles, we co-transfected HEK293 cells in T75 matrices with three psPAX2 (10 µg), ph-SΔ18 (10 µg), and pLeni-Luc-GFP (10 µg) plasmids in a 1:1:1 ratio. Lipofectamine 3000 (2 μL per μg of plasmid) (ThermoFisher, USA) was used as a transfectant. The transfected HEK293T cells were incubated at 37 °C in an atmosphere of 5% CO2 for 2 days, after which the supernatant containing lentivirus particles coated with SARS-CoV-2 protein were collected and filtered through a 0.45 μm filter (Millipore, USA), and then concentrated on the sucrose pillow. After concentration, 500 µL aliquots were made and stored at −80 °C.

##### Determination of Cytotoxicity of Compounds on HEK293T Cells

To determine the cytotoxic concentration of compounds, the day before compounds were added to 96-well culture plates, HEK293T cells were seeded in an amount of 100 μL cell suspension per well (104 cells per well), and placed in a CO_2_ incubator. The next day, after 24 h of incubation, different concentrations of test compounds were added to the cell culture by sprouting (initial concentration of 1 mg/mL). Each concentration was tested in three replicates. DMSO, at a concentration of no more than 1%, was added to the control wells. The final volume of medium in the well was 200 µL. The plate with added compounds was incubated in a CO_2_ incubator for 72 h at 37 °C and 5% CO_2_. After 72 h of incubation of the cell line with the tested compounds, 20 µL of MTT working solution (5 mg/mL) was added to each well and incubated for another 2 h under CO_2_ incubator conditions. After 2 h, plates were removed from the CO_2_ incubator, and the medium in each well was replaced with DMSO solution (50 µL/well). The plates were gently shaken to dissolve the formazan crystals. The optical density of each well at 570 nm was determined using a plate reader. The survival of HEK293T cells in the presence of the test substance was calculated using the formula: (OD of experimental wells − OD of medium)/(OD of control wells − OD of medium) × 100%, where OD is the optical density. The concentration causing 50% cell death (CC50) was determined from dose–dependent curves using GraphPad Prism 6 software (Ver. 6.04). For each compound tested for antiviral activity, a range of non-toxic concentrations was selected. A range of nontoxic concentrations, where antiviral activity was investigated, was chosen for each compound.

##### Pseudoviruses Neutralizing Assay

To determine the neutralizing capacity of the tested compounds, a neutralization assay was performed using 293-hAce2-TMPRSS2 cells (transient) and lentiviral particles exhibiting S protein of SARS-CoV-2. Briefly, serial dilutions of the compounds in DMEM culture medium (without serum or antibiotic) were prepared in 96-well plates. Then, suspension of pseudoviruses (10 μL/well) was added to the diluted compounds, and the mixture of compounds with pseudoviruses was incubated in a CO_2_ incubator for 1 h at 37 °C and 5% CO_2_. After 1 h, 293-hAce2-TMPRSS2 cells (transient) (1.5 × 104 cells/well) were added and incubated at 37 °C in 48 h. The assay was performed in two replicates. The infectivity of pseudoviruses in the presence of compound samples was determined by the luminescence index 48 h after infection. The percentage of neutralization of each sample was calculated as the ratio between the RLU values of the test wells (test sample + pseudovirus + cells) and the virus control (pseudovirus + cells).

### 2.3. Molecular Modeling

All theoretical calculations were carried out using software Schrodinger Small Molecule Drug Discovery Suite 2022-1 [31].

#### 2.3.1. Protein and Ligand Preparation

The geometric parameters of proteins were downloaded from the uncommercial database Protein Data Bank [32]. The full-size surface protein of SARS-CoV-2 (PBD code 7BNM [33]), the NTD complex of S-protein with biliverdine (7B62 [34]) and the main protease MPRO (7L0D [35]) were selected for molecular modeling. Model protein structures were prepared using Schrodinger Protein prepwizard tools: hydrogen atoms were added and minimized, missing amino acid side chains were added, bonds multiplicity was restored, solvent molecules were removed, and the entire structure was optimized in the OPLS4 force field [36].

The geometric parameters of the ligands were optimized by the force field method, considering all possible conformations. Compound **13** can exist in two isomers by the double C = C bond: Z and E. To determine the most stable isomer, quantum-chemical calculations were carried out in gas phase approximation by the DFT M05-2X method [37] supplemented by the dispersion correction GD3 [38] with the basic set of TZVP [39].

#### 2.3.2. Analysis of a Potential Binding Site

For docking into SARS-CoV-2 Mpro, the active site of the enzyme, containing catalytic amino acids Cys145 and His41, and located in a cleft between the two N-terminal domains I and II of the Mpro monomer, was chosen [40].

Geometric parameters of the S-protein with an entry inhibitor, with proven antiviral properties against SARS-CoV-2 are not available in the non-commercial database Protein Data Bank. This greatly complicates the search for a binding site for entry inhibitors. The possible binding sites were considered: the binding site of Arbidol [41,42] located in the region of heptad repeats (HR1), the region of the likely binding of UA-30 [43] and nelfinavir [44], located at the boundary of the two subunits of protein, and the NTD cavity [34]. Using the Phase plugin [45] by Schrodinger Suite software, the pharmacophoric profile of binding sites was described.

#### 2.3.3. Molecular Docking Procedure

Docking to the SARS-CoV-2 Mpro performed the function of primary virtual screening of the possible mechanism of action for all synthesized compounds and was carried out in comparison with the ML188 inhibitor.

Compounds that showed activity against the pseudoviral system in biological tests were selected to assess affinity to S-protein., Compound **14** with similar structural descriptors to active compound **3** was considered as a negative control for docking.

Molecular docking was performed using a forced ligand positioning protocol (Glide induced fit docking or Glide IFD with the following conditions: flexible protein and ligand, 15 Å grid matrix size, and amino acids within 5 Å of the ligand were constrained to be optimized for ligand influence. Docking solutions were ranked by evaluating the following calculation parameters: docking score (based on GlideScore with penalties exclusion), ligand efficiency (LE, where the per-heavy-atom distribution of the scoring function is considered), and the model energy value parameter (Emodel), including GlideScore value, energy of unbound interactions, and energy parameters spent on the formation of compound stacking at the binding site.

## 3. Results and Discussions

### 3.1. Synthesis of Usnic Acid Derivatives

**Scheme 1:**
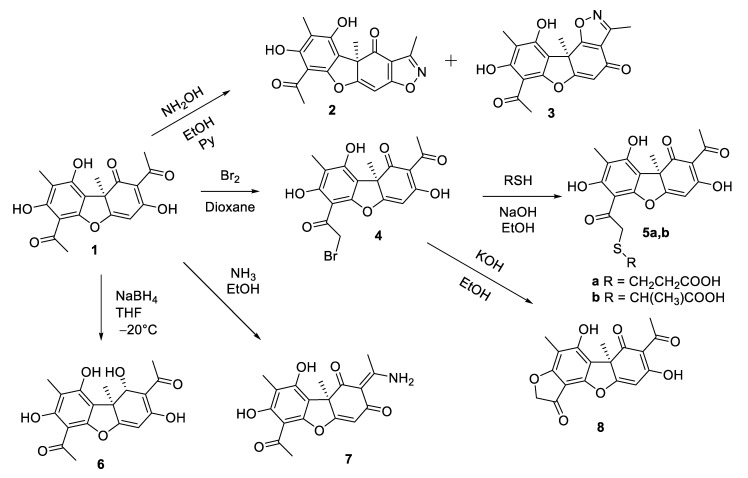
Synthesis of compounds **2**–**8** based on (+) unic acid **1**.

Commercially available (+)-usnic acid **1** was used as a starting compound. Based on the favorable prognosis in silico for usnic acid as a ligand for SARS-CoV-2 targets [19,20], we chose to synthesize a range of its derivatives, not significantly different from parent compound in volume and differing in the localization of chemical modifications: the triketone system of the C ring, the acetyl group of the A ring, and the C4-C4a double bond. Derivatives with the isoxazole cycle **2** and **3** were obtained by the reaction of (+)-usnic acid with hydroxylamine in ethanol (Scheme 1) [23]. The bromination of usninic acid yielded a bromine derivative **4**, which then reacted with thiols to give thioesters **5a,b** [25]; compound **5b** is a mixture of diastereomers (1:1) and has not been described previously. Derivative **6** was prepared by reduction of (+)-usnic acid with sodium borohydride in THF under cooling [26]. The reaction of (+)-usnic acid with ammonia in ethanol yielded the compound **7** [27]. Furanone **8** was obtained by treating the bromine derivative **4** with bases in ethanol [28].

**Scheme 2:**
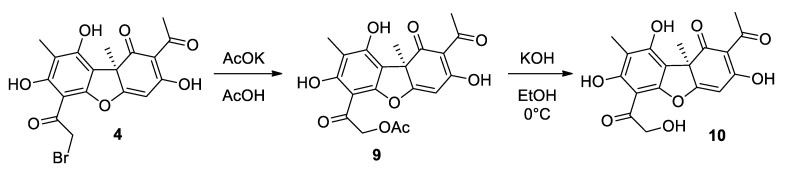
Synthesis of compound **10** based on bromoderivative of usnic acid **4** via acelated product **9**.

Compound **10** was obtained by a two-step synthesis (Scheme 2). At the first stage the bromoderivative of usnic acid **4** reacted with potassium acetate in acetic acid [24]. The obtained acetate **9** was transferred without purification into the reaction of hydrolysis with potassium hydroxide in ethanol in an ice bath. As a result, after purification by column chromatography, compound **10** was obtained in 62% yield.

**Scheme 3:**
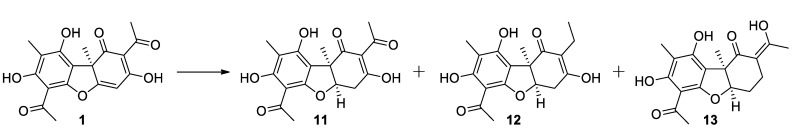
Hydrogenation of usnic acid **1** leading to products **11–13**.

A series of compounds **11**–**13** was obtained using a modified method of hydrogenation of usnic acid (Scheme 3). For this purpose (+)-usnic acid **1** was placed with a catalyst (Pd/C 10%) in THF and incubated in a hydrogen atmosphere at room temperature for 24 h. After separation by column chromatography, compounds **11**–**13** were isolated with yields of 35%, 36%, and 15%, respectively.

**Scheme 4:**
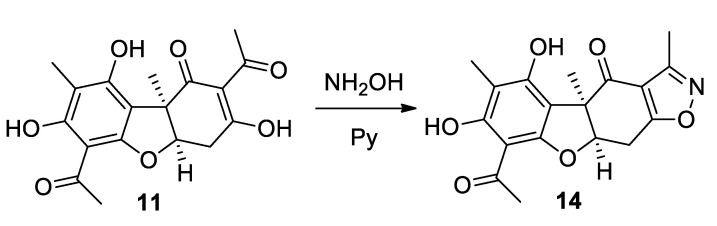
Synthesis of isoxazole **14** based on dihydrousnic acid **11**.

By adding hydroxylamine to the mixture of dihydrousnic acid **11** with pyridine, the previously undescribed isoxazole **14** was obtained and isolated using column chromatography in 54% yield (Scheme 4).

Thus, twelve derivatives of (+)-usnic acid modified at different positions of the dibenzofuran backbone were obtained for antiviral activity tests: by modification of the substituent in cycle A (compounds **5a**,**b**, **8**, **10**), reduction of double bonds and functional groups of cycle C (compounds **6**, **11**–**13**), and derivatization of functional groups of cycle C of (+)-usnic acid (compounds **2**, **3**, **7**, **14**). The structure of the obtained compounds was established on the basis of ^1^H and ^13^C NMR spectra and high-resolution mass spectra.

### 3.2. Study of Antiviral Activity

#### Biological Testing for an Infectious Virus

The compounds synthesized in this work were tested using SARS-CoV-2 infectious viruses. Testing on an infectious virus allows us to understand whether the compound has activity against the specified virus, but it does not give an understanding of the mechanism of action of this agent. Thus, our initial screening was performed on Vero cells using the SARS-CoV-2 virus strain Wuhan – hCoV-19/Australia/VIC01/2020 line B. Viruses of this genetic line circulated at the beginning of the first wave of the pandemic, and are now considered prototypic [46]. The studies were performed in a BSL-3 biosafety level laboratory at the State Research Center “Vector”. The concentrations of 50% inhibition (IC_50_), 50% toxic concentration (CC_50_), and SI selectivity index were determined for the studied compounds. The results are shown in Table 1. We used remdesevir as a reference drug. The studies showed that the initial compound, (+)-usnic acid **1**, exhibited pronounced activity against the SARS-CoV-2 strain of Wuhan virus with an IC_50_ of 10.9 μM, while possessing relatively low toxicity. The biological properties of isoxazole derivatives **2** and **3** differ dramatically. Thus, agent **2**, which has an isoxazole fragment in the 2–3 positions of the usnic backbone, was less toxic than the original usnic acid, but did not exhibit antiviral activity at all. Agent **3**, in which the isoxazole cycle is condensed with 1–2 positions of the natural backbone, showed high activity against the SARS-CoV-2 virus, but this compound was significantly more toxic than the original usnic acid. Compounds **5a,b** modified with thioacids at position 14 of the usnic acid have comparable toxic properties with the original molecule, but have either no antiviral activity at all as agent **5a**, or low activity as agent **5b**. Compound **6**, which has a reduced keto group in the 1 position of the backbone, also exhibits moderate activity, but its toxic properties are higher than those of the natural usnic acid. Agent **7**, which has a primary amino group in cycle C, has no antiviral activity at all in our experiments, but is significantly less cytotoxic than the parent substance. Modification of cycle A with the formation of a furanone fragment in agent **8,** also, does not lead to an increase in the target activity; IC_50_ of this substance lies in the average micromolar range. Compound **10,** having an additional hydroxyl group in the 14 position of the usnic acid, shows less cytotoxicity on the studied cell line and pronounced activity against the Wuhan strain of the SARS-CoV-2 virus, and the selectivity index of this agent SI is 12. Among compounds **11**–**13** having a reduced C-cycle fragment, agent **13** shows the highest activity. Thus, the IC_50_ of this substance lies in the lower micromolar range, and agent **11** also showed pronounced activity. Compounds **11** and **13** are somewhat more toxic on the studied cell line, but deserve special attention as potential antiviral agents. Isoxazole derivative **14**, which has a structure similar to agent **2**, but reduced by the C cycle, does not exhibit antiviral properties, just like isoxazole **2**.

For compounds that showed activity, testing was additionally performed using two more virus strains, Delta and Omicron. Thus, after the Wuhan strain spread around the world, many unique mutations in the virus genome were registered. The biological advantages provided by some of these mutations led to the formation of certain genetic lines that greatly outperformed the original strain. Lines that are characterized by increased transmissibility or virulence or that reduce the effectiveness of available countermeasures are classified by the WHO as variants of concern. One such strain, Delta variant hCoV-19/Russia/PSK-2804/2021, lineage B1.617.2, was first detected in India in June 2021. This variant of the virus quickly became the predominant lineage worldwide due to its much higher transmissibility. Three distinctive features of this strain are known: increased infectivity, enhanced ability to bind to lung cell receptors, and potential resistance to monoclonal antibody therapy. The Delta variant is associated with increased disease severity, as evidenced by the higher rate of hospitalization among patients with Delta COVID-19 as compared to the alpha variant, especially among unvaccinated individuals [47]. The Omicron strain of SARS-CoV-2 (B.1.1.529)—first identified in Botswana and South Africa in November 2021—has a large number of mutations in the surface protein. Omicron virus strains are responsible for a significant increase in incidence worldwide in late 2021 and January–February 2022 [48,49].

For extended testing, we chose usnic acid, isoxazole derivative **3**, furanone **8**, hydroxy derivative **10**, and C-ring reduced agents **11**–**13**. The results of biological testing showed that usnic acid exhibited a wide range of antiviral activity. Thus, the activity against the Omicron strain is comparable to that of the comparison drug remdesevir, while the activity against the Delta strain is slightly lower than that against the prototypical Wuhan strain. The activity of agent **3** is also higher on the Omicron strain than on the Wuhan and Delta strains. Furanon **8** had almost no activity on the Delta strain; the activity on Omicron was comparable to that in the primary test. Reduced compound **13** showed high activity against all three strains. We directed our further efforts to the study of the mechanism of antiviral action.

### 3.3. Investigating the Mechanism of Action

Current trends in the search for new antiviral agents suggest that it is optimal to affect the virus, either during the intracellular life cycle or at an early stage of virus entry into the cell [4]. In this case, the key targets of SARS-CoV-2 replication inhibitors can be the main viral protease or the surface protein responsible for virus entry.

#### 3.3.1. Main Viral Protease 3CLPro as a Target of Activity

Usnic acid belongs to dibenzofuran derivatives, and is formally a polyphenolic compound. High activity against the main viral protease 3CLPro was previously shown for substances of this class. Thus, it was described that flavanoids—plant polyphenols Scutellarein, Dihydromyricetin, Quercetagetin, and Myricetin—are active against 3CLPro at a dose of 1.2–5.8 μM [50]. It has recently been shown that Scutellarein methylated at the 4′ position exhibits activity against the main protease at submicromolar concentrations [51]. In addition, it is known that the phenolic groups of polyphenols can be converted into orthoquinone under oxidizing conditions, which can be easily attacked by nucleophiles (e.g., the S-H group of the main protease) [52]. The covalent mechanism of action of lavonoids on 3CLPro of this type is suggested by the authors of [53]. At the same time, such a mechanism of action is unlikely for usnic acid and its derivatives because of its structural features.

In an attempt to study the possible mechanism of antiviral action, which was revealed for a number of compounds in experiments on live virus, we performed molecular modeling of the interaction of compounds with the active site of the main protease of SARS-CoV-2. Given the known reactivity of usnic acid [54,55], it was assumed that covalent binding of the SH-group of the catalytic amino acid residue Cys145 is unlikely. Therefore, non-covalent docking to the active site of the main protease was performed. A docking evaluation function showed that for several new usnic acid derivatives, the minimum binding energy was comparable with that of the model inhibitor ML-188 (Table 2).

**Figure 2:**
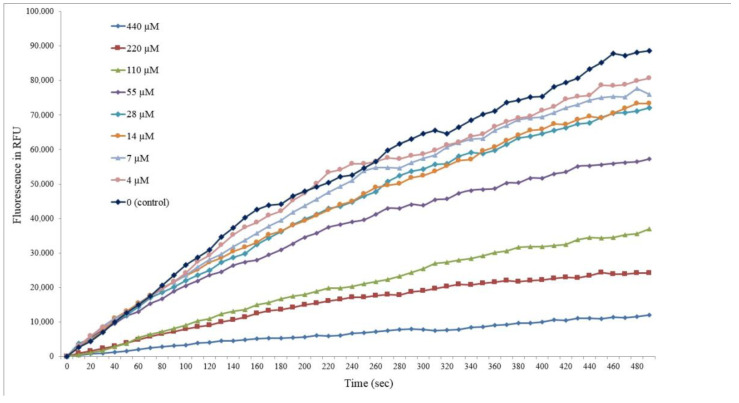
Fluorescence change depending on inhibitor concentration agent **6**.

Taking into account the calculated data obtained, we performed an experimental evaluation of the inhibitory activity of (+)-usnic acid and its derivatives against the main protease of SARS-CoV-2 by the FRET method (Fourier resonance transfer of fluorescence energy method). Validation of the system developed by us was performed using the drugs Disulfiram (IC_50_ 6.1 ± 0.6 μM) and Ebselen (IC_50_ 1.7 ± 0.46 μM) for comparison. Several compounds showed weak inhibitory activity as a result of the experiment: **6** (IC_50_ 111.75 μM), and **7**, **8**, and **10** (IC_50_ about 200 μM). Figure 2 shows the change in fluorescence depending on the concentration of inhibitor agent **6**.

**Figure 3:**
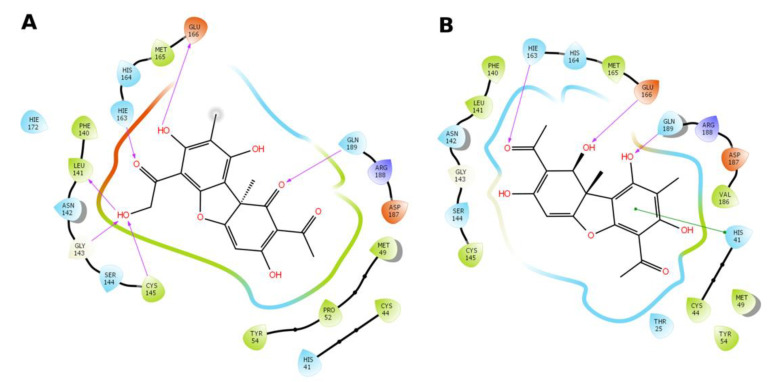
Noncovalent interactions of usnic acid derivatives at the active site of the main protease of SARS-CoV-2. (**A**)—agent **10** and (**B**)—agent **6**. Noncovalent interactions of co-compounds at the active site are shown with arrows: purple—hydrogen bonds and green—stacking interactions.

Apparently, compounds **10** and **6** can be weak inhibitors of the main SARS-CoV-2 protease. According to the authors of the X-ray structural model of the non-covalent interaction between the ML188 inhibitor and the main SARS-CoV-2 protease, its inhibitory activity is associated with the stabilization of the flexible loop structures of the main protease active site at the 141–145 chain region and the formation of non-covalent interactions with amino acid residues Cys145 and His41 that perform the catalytic function of the enzyme [35]. Usnic acid derivatives do not possess the same branched molecular structure as ML188, adapted for interaction with the deep pockets of the main protease active site. However, the abundance of polar groups attached to the polycyclic core of the usnic acid allows the new derivatives to quite successfully form hydrogen bonds in the central part of the active site of the main protease of SARS-CoV-2 (Figure 3). Compound **10** (Figure 3A) can theoretically form hydrogen bonds with Leu141, Gly143, Glu166, Gln189, and the catalytic amino acid residue Cys145. In the case of compound **6** (Figure 3B), the possible formation of hydrogen bonds with Gly143, Hie163, and Gln189 is observed. The aromatic systems of the benzyl cycle of derivative **6** and Hys41 can form a stacking interaction. The probability of the formation of intramolecular hydrogen bonds between the hydroxyl groups of the derivatives, which can affect their rotation angles, is apparently high.

It is important to note that the initial usnic acid **1** in our studies is also not an inhibitor of the above protease, despite the fact that earlier articles based on molecular modeling suggested a high potential of usnic acid as an inhibitor of 3CLpo [19].

#### 3.3.2. Study of Early Viral Replication

##### Testing with the Use of Pseudo-Viral Systems

To find out whether the usnic acid derivatives are virus entry inhibitors, additionally synthesized compounds were tested in a neutralization assay using pseudoviruses bearing S glycoprotein S of the SARS-CoV-2 virus on their surface. In this study, HEK293T-hAce2-TMPRSS2 (transient) cell lines were used according to the methodology described in [56]. We used Arbidol as a reference drug.

We studied the toxicity of the compounds on the mentioned cell line, and studied the percentage of neutralization of pseudovirus particles under the action of substances at a dose of 25 μM (Table 3). Substances **2**, **3**, **11**–**13** were shown to be active against the pseudovirus system. Thereafter, a more revealing IC_50_ was performed for the agents that showed the highest activity. According to the results presented in Table 2, compound **13** showed the greatest activity, for which the IC_50_ was 5.28 μM with SI 28. These data are in agreement with the results obtained on infectious viruses (Table 1), and indirectly indicate that the potential target of this compound is the glycoprotein S of the SARS-CoV-2 virus. Another compound to watch out for is agent **11** with an IC_50_ of 22 μM and SI 21.

It is interesting to note that despite the structural similarity of the usnic acid derivatives, their activities against the pseudoviral system differ significantly. Compounds **5a** and **8** showed no activity against the pseudovirus system at all, and the original usnic acid, which showed moderate activity on three SARS-CoV-2 virus lines, is not an entry inhibitor.

##### Competitive Inhibition of the RBD/ACE2 Interaction Based on ELISA

Compounds capable of inhibiting the entry of pseudoviruses into target cells can act through various mechanisms. This can be direct blocking by substances of the binding of the virus surface proteins to their target receptors. Indirect inhibition is also possible, when substances block conformational rearrangements or other important steps in the work of viral fusion proteins. The implementation of the first blocking mechanism is quite easy to detect if the virus has a single target for interaction. For SARS-CoV-2, this is possible because the target of this virus is the ACE2 cell receptor. For the compounds studied in this work, we analyzed their ability to inhibit the interaction of recombinant ACE2 with RBD. It was shown that none of the compounds used in this work are capable of inhibiting this interaction, so we assume that the compounds studied have an inhibitory effect on the second pathway.

### 3.4. Molecular Modeling Study

#### 3.4.1. Pharmacophore Profile of Entry Inhibitor Binding Sites

**Figure 4:**
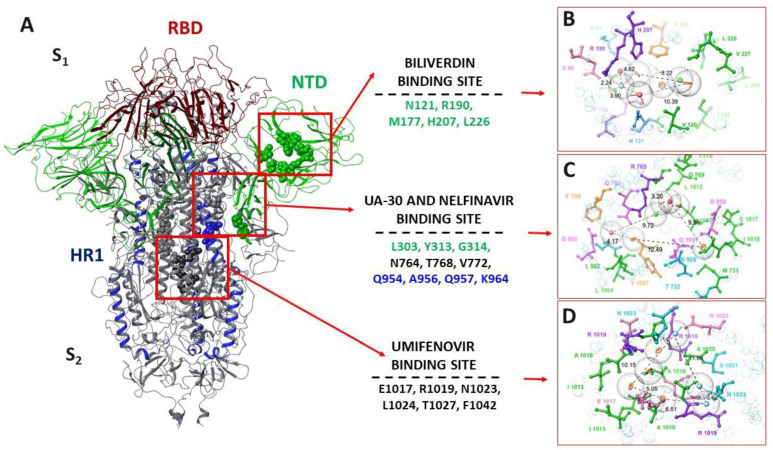
(**A**)—Secondary structure of the SARS-CoV-2 surface protein. Visualization based on the PBD code 7BNM [33]: the secondary structure of the N-terminal domain (amino acids 14–330) is shown in green; the receptor-binding domain (amino acids 330–528) is shown in dark red; heptad repeats (amino acids 910–985) are shown in blue. (**B**–**D**)—Pharmacophore profile of inhibitor binding sites. The dots indicate the required pharmacophoric features of the potential ligand: green ball corresponds to the hydrophobic feature; blue and pink correspond to the donor and acceptor, respectively; orange indicates aromatic rings; red indicates negatively charged. The distances between the pharmacophore features are indicated in Å. Amino acids are shown within 4 Å of the likely location of the ligand. Neutral hydrophobic aliphatic amino acids are colored green; basic hydrophilic amino acids are colored purple; neutral hydrophilic amino acids are colored blue; neutral hydrophobic aromatic amino acids are colored orange; hydrophilic acidic amino acids are shown in pink.

Surface glycoprotein S is a type I transmembrane fusion protein with a mass of 180 to 200 kDa. The N-end of the protein faces the extracellular space and is retained in the viral membrane through a transmembrane domain with a short C-terminal segment facing the intracellular space. Structural modeling of the spike protein shows that S1 and S2 subunits form the bulbous head and stem region, respectively. The S1 subunit (amino acids 14 to 816) contains two subdomains, the N-terminal domain (NTD—amino acids 14–528) and the C-terminal domain (CTD—amino acids 529–686). A fragment of the subdomain forms the receptor binding domain (PCD—amino acids 331–528). The S2 transmembrane region (816 to 1234 amino acids) contains domains involved in the fusion of the viral and cell membranes. These include the fusion peptide and two heptad repeats, HR1 (910–985) and HR2 (1163–1210) (Figure 4A). The HR domains consist of α-spirals and, as a rule, their position and amino acid sequence are conservative for the entire coronavirus family [57].

Despite a detailed study of the structure and function of the surface protein, the binding site of potential entry inhibitors is still quite controversial. The geometrical parameters of the full-length spike protein in the database are present in different conformations, for different strains, and/or bound to antibodies. However, the structures of the S-protein or its part (e.g., RSD) in the complex with the ligand are still missing, despite the fact that the pandemic has been ongoing since 2020. This fact, of course, complicates the search for a potential binding site for entry inhibitors. Based on the data from the biological experiment on the pseudovirus system and the lack of activity in the protein–protein interaction system (RBD-ACE), we assume that the active compounds can bind in any of the protein surface cavities other than RBD. Then, the following spike–protein cavities can be considered as potential binding sites with an assessment of their pharmacophore profile. First, the NTD complex with biliverdin, a green bile pigment, was considered. The site is saturated with hydrophobic amino acid residues (Figure 4B) as well as hydrophilic amino acids, such as arginine and histidine, that are prone to donor-acceptor interactions with the ligand. According to the studies of the authors of [34], biliverdin tightly adheres to the binding pocket with the formation of a number of molecular interactions with the side chains of residues Asn1121, Arg1190, and His1207. Binding site analysis suggests that the potential ligand should contain aromatic rings, at least one hydrophobic fragment, groups capable of donor-acceptor interactions, and the presence of a negatively charged fragment is desirable.

The second binding site was determined based on the results of molecular modeling and data from biological experiments to create a UA-30 inhibitor-resistant strain of the virus, published in [43,44]. The region between the two subdomains, closer to HR1, contains hydrophobic and aromatic amino acids (Figure 4C). For pronounced affinity, the ligand must be sufficiently bulky, contain aromatic rings, an acceptor group, a negative charged fragment, and a hydrophobic fragment. Finally, the binding site of Umifenovir (Arbidol) in the region of the central heptads was also examined by us [41,42]. The site contains hydrophilic basic amino acids, amino acids capable of donor interactions, and hydrophobic residues (Figure 4D). The binding site was chosen on the basis of the small antifluorescent activity of the derivatives of usnic acid. To fit tightly into the binding pocket, the potential ligand must essentially consist of a set of aromatic rings and hydrogen bond donor groups.

#### 3.4.2. Molecular Docking Procedure

The molecular docking procedure was performed for compounds (**2**, **3**, **11**–**13**) that showed activity in pseudovirus tests. Inactive compound **14** was considered as a negative control. Analysis of the results of molecular docking (energy parameters, visualization of intermolecular contacts, and correspondence with the pharmacophoric profile of the potential ligand) suggests that the binding site of Biliverdin can be considered as the binding site of the usnic acid derivatives. First, the pharmacophore features of an “ideal” ligand are characteristic of all compounds (3 to 4 among 7 possible); namely, aromatic rings, a hydrophobic fragment, and a hydrogen bond acceptor. Second, the arrangement of the active compounds (**11**–**13**) lacks the penalty “clash” interactions. Finally, the leader compound **13** is characterized by the lowest values of energy parameters, such as glide score and Emodel, while the inactive compound **14** has the highest values (table with energy parameters and ligand locations in the active site, presented in the SM).

Usnic acid derivatives can also bind in the heptadic region. However, this site is larger, and ligand binding in it is likely to be short-lived. Only two compounds, 2 and 3, have more than two out of six possible pharmacophore features. However, the significant activity of these compounds in tests using the pseudovirus system is not confirmed. In addition, there is no pronounced dependence in the binding of ligands in this site on the data of a biological experiment. The location of the ligands differs from the binding of UA-30 and nelfinovir described in [43,44]. The reason is the size of molecules. The bulk molecule UA-30 covers the entire surface of the site, contacting amino acids from both subunits. Smaller derivatives of the ascanoic acid bind between the spirals of HR1 of the second subunit. Binding of compounds at the Arbidol binding site is unlikely. This is evidenced by the pharmacophore profile of the site and energy characteristics (see SM).

**Figure 5:**
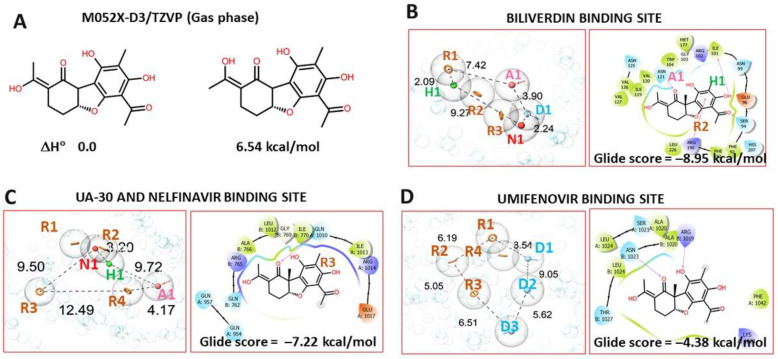
Results of quantum chemical calculations and molecular docking procedure agent **13** into probable binding sites: (**A**)—two possible isomeric form E and Z of lead-compound **13**; (**B**–**D**)—the locations of **13** in bindig site of S-protein.

The leader compound **13** can be in two possible isomeric forms E and Z. The binding of the isomers at the binding sites can differ. Methods of quantum chemistry were used to estimate the difference in enthalpy of the two isomers, and showed that the E-isomer is at least 6 kJ/mol more stable than the Z-isomer (Figure 5A). For this reason, molecular docking analysis was performed only for the E-isomer. The location of agent **13** in the biliverdin binding site is characterized by the formation of hydrogen bridges between the oxygen atoms of agent **13** and the amino acids Ile101, Asn121, and Ser94. π–cation stacking interaction is observed between the aromatic ring and Arg190. Three pharmacophore features out of seven are present in the ligand (Figure 5B). In the heptad repeats region (HR1), **13** forms only a hydrogen bridge with Gln1080 (Figure 5C). The presence of the R3 pharmacophoric feature in the ligand is not realized during binding. The location of agent **13** at the Umifenovir binding site is characterized by the formation of hydrogen bridges with Arg1019 and Asn1023 and high energy values.

Thus, based on the analysis of the results of molecular modeling, we can assume that usnic acid derivatives bind in the N-terminal domain at the binding site of the hemoglobin decay metabolite. The function of the N-terminal domain of the surface protein is not well understood [34]. However, there is a clear relationship between RBD and NTD. Blocking NTD can reduce the reactivity of the S-protein SARS-CoV-2 and make it difficult for RBD to contact ACE2.

## 4. Conclusions

As a result of this study, we synthesized twelve derivatives of (+)-usnic acid modified at different positions of the dibenzofuranic base: by modifying the substituent in cycle A (compounds **5a**,**b**, **8**, **10**), derivatives containing the reduced cycle C (compounds **6**, **11**–**13**), and derivatives containing a nitrogen atom at cycle C of (+)-usnic acid (compounds **2**, **3**, **7**, **14**). Initial (+)-usnic acid **1** showed pronounced activity against three strains of the SARS-CoV-2 virus (Wuhan, Delta, and Omicron lineages), with the lowest IC_50_ of the natural compound being shown against the Omicron strain (3.7 μM, SI 39). Addition of the isoxazole ring or amination of the natural backbone leads to loss of antiviral activity. Modification of usnic acid to obtain thioesters at the 14th position also does not lead to an increase in antiviral activity. The input of additional hydroxyl groups or reduction of the C-cycle double bonds resulted in agents that also have high activity against three strains of the virus.

Our biological studies of the mechanism of action show that usnic acid and its derivatives in our experiments are not inhibitors of the main viral protease, or show weak activity. Our experiments using a pseudovirus system with glycoprotein S on the surface of the SARS-CoV-2 virus showed that usnic acid is not an inhibitor of virus entry, whereas its derivatives **2**, **3** and **11**–**13** exhibits pronounced inhibitory activity. Thus, the IC_50_ on the pseudovirus system for agent **13** is 5.3 μM. The data correlate with the experiment on the infectious virus. An experiment using protein–protein interaction (RBD-ACE) allowed us to exclude the RBD site of the surface protein. We considered three binding sites in the glycoprotein and performed a molecular docking procedure for compounds (**2**, **3**, **11**–**13**) that showed activity in pseudovirus tests. Based on the analysis of the molecular modeling results (Supplementary Materials Table S1) and the obtained biological data, we can assume that the usnic acid derivatives bind in the N-terminal domain at the binding site of the hemoglobin decay metabolite. At the same time, the mechanism of the antiviral action of natural (+)-usnic acid is still a challenge.

## Figures and Tables

**Table 1:** Results of study of antiviral activity of compounds against SARS-CoV-2 viruses.

Agent		Wuhan Lineages B ^a^		Delta Lineage B.1.617.2 ^b^		Omicron Lineage B.1.1.529 ^c^	
	CC_50_^d^_,_ µM	IC_50_^e^, µM	SI ^f^	IC_50_, µM	SI	IC_50_, µM	SI
**1**	146.7 ± 22.1	10.9 ± 2.2	13	20.9 ± 3.4	7	3.7 ± 0.8	39
**2**	252.2 ± 31.0	NA	-	NT	-	NT	-
**3**	15.5 ± 2.8	9.2 ± 2.4	1.6	9.1 ± 1.8	1.7	3.5 ± 1.1	4.5
**5a**	177.3 ± 18.2	NA	-	NT	-	NT	-
**5b**	176.5 ± 22.6	76.5 ± 8.2	2	NT	-	NT	-
**6**	99.0 ± 12.1	65.3 ± 7.4	1,5	NT	-	NT	-
**7**	527.7 ± 16	NA	–	NT	-	NT	-
**8**	175.8 ± 19.9	44.3 ± 5.2	4	95 ± 8.7	2	47.0 ± 6.5	4
**10**	306.3 ± 28.6	25.3 ± 2.1	12	9.7 ± 1.4	31	17.5 ± 3.8	17
**11**	84.9 ± 12.1	21.0 ± 3.5	4	NA	-	NA	-
**12**	262 ± 32.0	91 ± 7.1	3	85 ± 11.2	3	60 ± 12.1	4
**13**	72.7 ± 14.6	6.7 ± 1.4	10	13.5 ± 2.1	5	6.6 ± 1.9	10
**14**	295.5 ± 11.9	NA	-	NT	-	NT	-
Remdesivir	710.9 ± 21.2	3.8 ± 0.42	186	2.1 ± 0.16	338	2.0 ± 0.13	356

^a^ hCoV-19/Australia/VIC01/2020 (EPI_ISL_406844); ^b^ hCoV-19/Russia/PSK-2804/2021 (EPI_ISL_7338814); ^c^ hCoV-19/Russia/Moscow171619-031221/2021 (EPI_ISL_8920444); ^d^ CC_50_ is the cytotoxic concentration, the concentration resulting in the death of 50% of cells; ^e^ IC_50_ is the 50% virus-inhibiting concentration, the concentration leading to 50% inhibition of virus replication; ^f^ SI is the selectivity index, the ratio of CC_50_/IC_50_. The data presented are the mean of three independent experiments. The values for CC_50_ and IC_50_ are presented as the mean ± error of the experiment.

**Table 2:** Results of the molecular docking of (+)-usnic acid and its derivatives into the active site of the main protease SARS-CoV-2 in comparison with the co-crystallized inhibitor ML188.

Ligand	Glide Score	Emodel	IFD Score
**10**	−11.568	−72.557	−664.659
**1**	−9.782	−67.242	−662.940
**6**	−9.408	−70.692	−663.973
**5b**	−9.300	−89.025	−662.874
**2**	−9.152	−49.980	−663.230
**12**	−9.029	−70.173	−662.796
**8**	−9.017	−73.237	−661.496
**3**	−8.736	−67.297	−663.686
**5a**	−8.619	−83.363	−665.003
**ML188**	−8.379	−83.299	−666.051
**14**	−7.768	−64.279	−660.219
**7**	−7.657	−67.654	−661.368
**11**	−7.563	−65.594	−660.863
**13**	−6.884	−53.540	−660.770

**Table 3:** Inhibitory activity against pseudoviruses pseudotyped with S protein of Delta lineage B.1.617.2.

Agent	CC_50_^a^, µM	% Neutralization at 25 µM	IC_50_^b^ µM	SI ^c^
**1**	>1000	12	**NT**	-
**2**	>1000	55	30 ± 2.9	33
**3**	150 ± 18.5	65	32 ± 5.1	6
**5a**	>1000	0	NT	-
**5b**	>1000	41	NT	-
**6**	168 ± 21.2	40	NT	-
**7**	850 ± 22.6	12	NT	-
**8**	358 ± 31.1	0	NT	-
**10**	750 ± 36.6	45	NT	-
**11**	469 ± 24.2	78	22 ± 3.2	21
**12**	>1000	53	28 ± 3.7	35
**13**	151 ± 14.9	95	5.28 ± 0.8	28
**14**	>1000	15	NT	-
**Arbidol**	19.8 ± 4.6	58	12.6 ± 4.1	2

^a^ CC_50_ is the cytotoxic concentration, the concentration resulting in the death of 50% of cells; ^b^ IC_50_ is the 50% virus-inhibiting concentration, the concentration leading to 50% inhibition of virus replication; ^c^ SI is the selectivity index, the ratio of CC_50_/IC_50_. The data presented are the mean of three independent experiments. The values for CC_50_ and IC_50_ are presented as the mean ± error of the experiment.

## Data Availability

Not applicable.

## Supplementary Materials

The following supporting information can be downloaded at: https://www.mdpi.com/article/10.3390/v14102154/s1, Figure S1: NMR 1H spectrum of **5b**; Figure S2: NMR 13C spectrum of **5b**; Figure S3: DFS spectrum of **5b**; Figure S4: NMR 1H spectrum of **10**; Figure S5: NMR 13C spectrum of **10**; Figure S6: DFS spectrum of **10**; Figure S7: NMR 1H spectrum of **12**; Figure S8: NMR 13C spectrum of **12**; Figure S9: DFS spectrum of **12**; Figure S10: NMR 1H spectrum of **13**; Figure S11: NMR 13C spectrum of **13**; Figure S12: DFS spectrum of **13**; Figure S13: NMR 1H spectrum of **14**; Figure S14: NMR 13C spectrum of **14**; Figure S15: DFS spectrum of **14**; Figure S16: Location ligands in the biliverdin binding site; Figure S17: Location ligands in the UA-30 and nelfinavir binding site; Figure S18: Location ligands in the umifenovir binding site; Table S1: Molecular modeling results.
