# Supplementary material for: (+)-Usnic Acid and Its Derivatives as Inhibitors of a Wide Spectrum of SARS-CoV-2 Viruses
Source: Viruses. 2022 Sep 29;14(10):2154. doi: 10.3390/v14102154 (PMC9611092; doi:10.3390/v14102154)
Supplement: Supplementary file 1 [file viruses-14-02154-s001.zip › viruses-1898780-supplementary.pdf]

viruses

MDPI

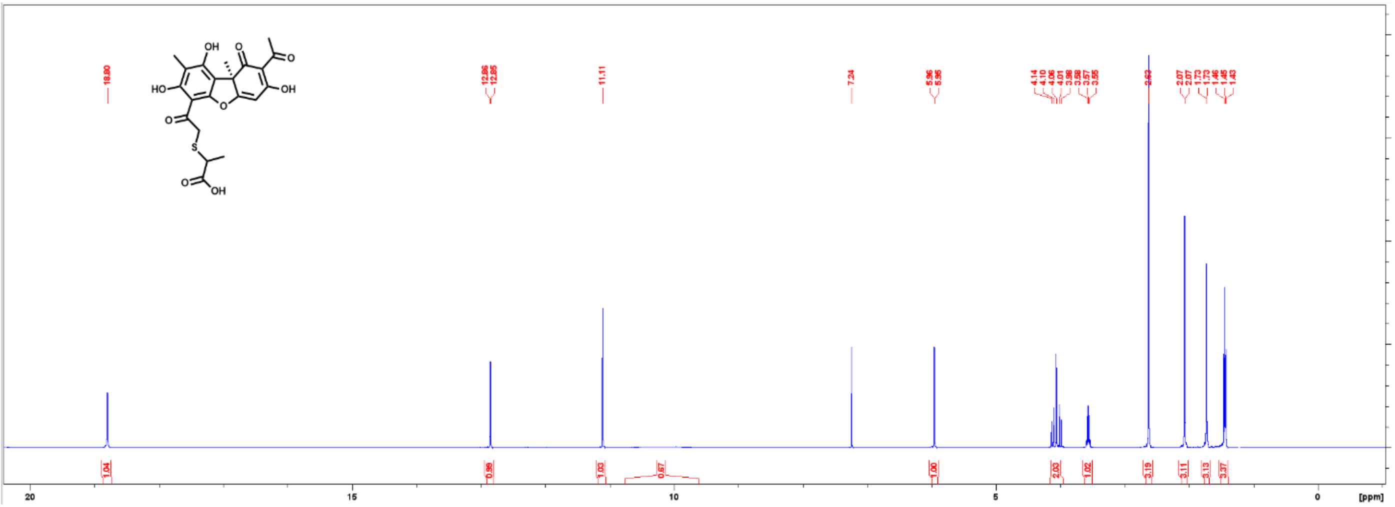

Figure S1. NMR ^{1}H spectrum of **5b.**

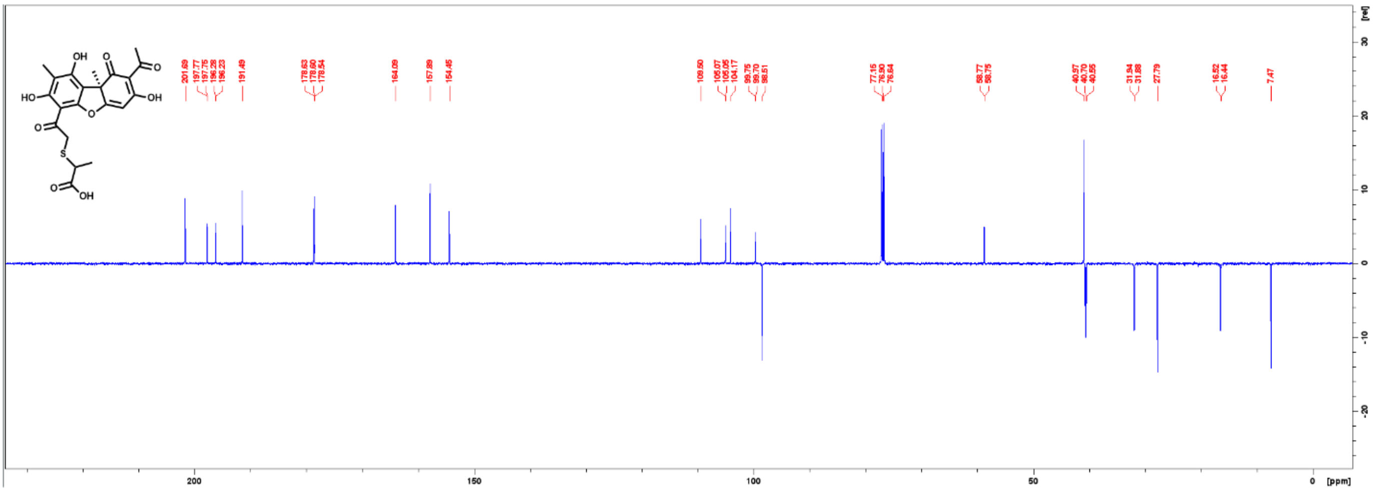

Figure S2. NMR ^{13}C spectrum of **5b.**

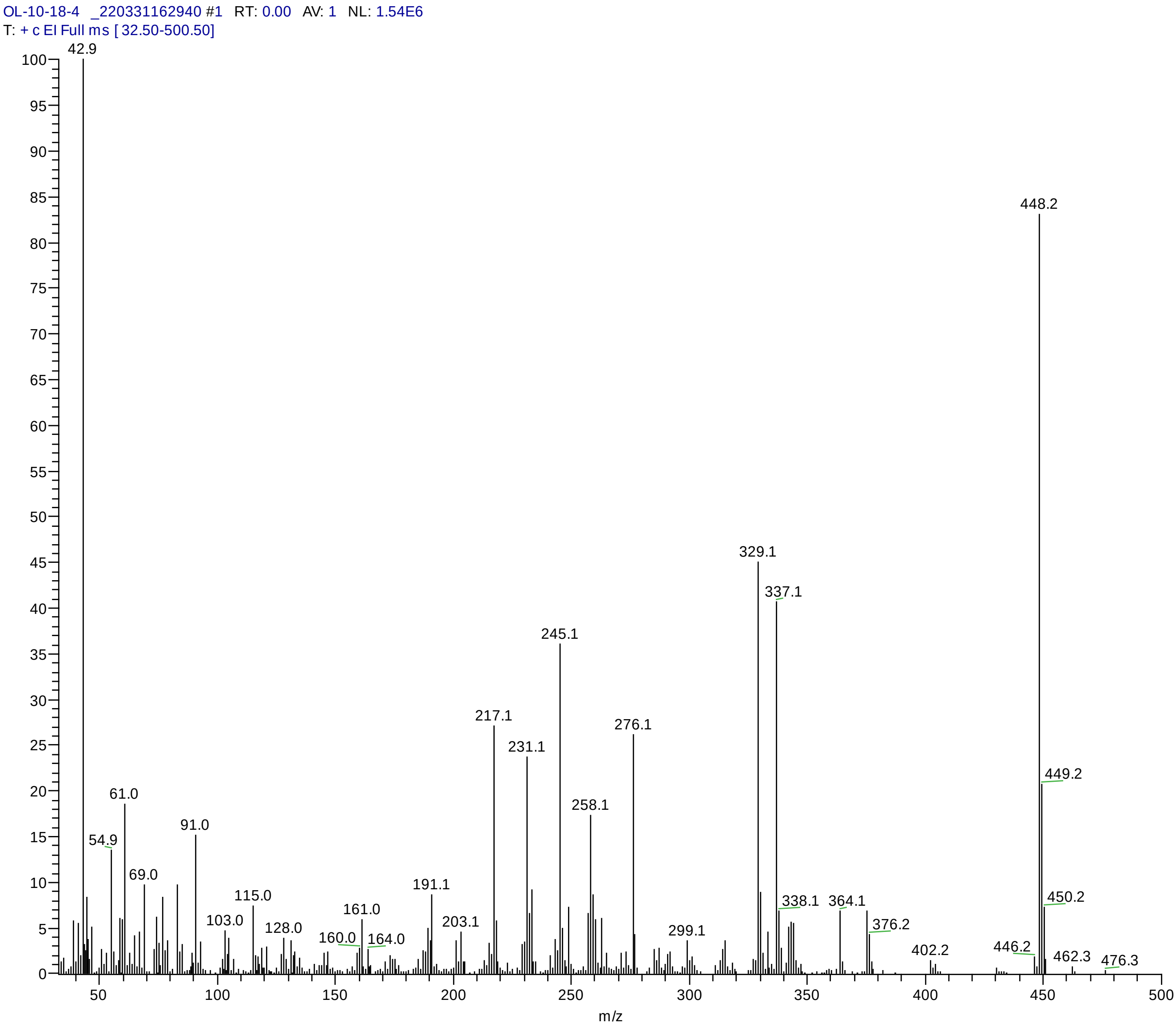

Figure S3. DFS spectrum of **5b.**

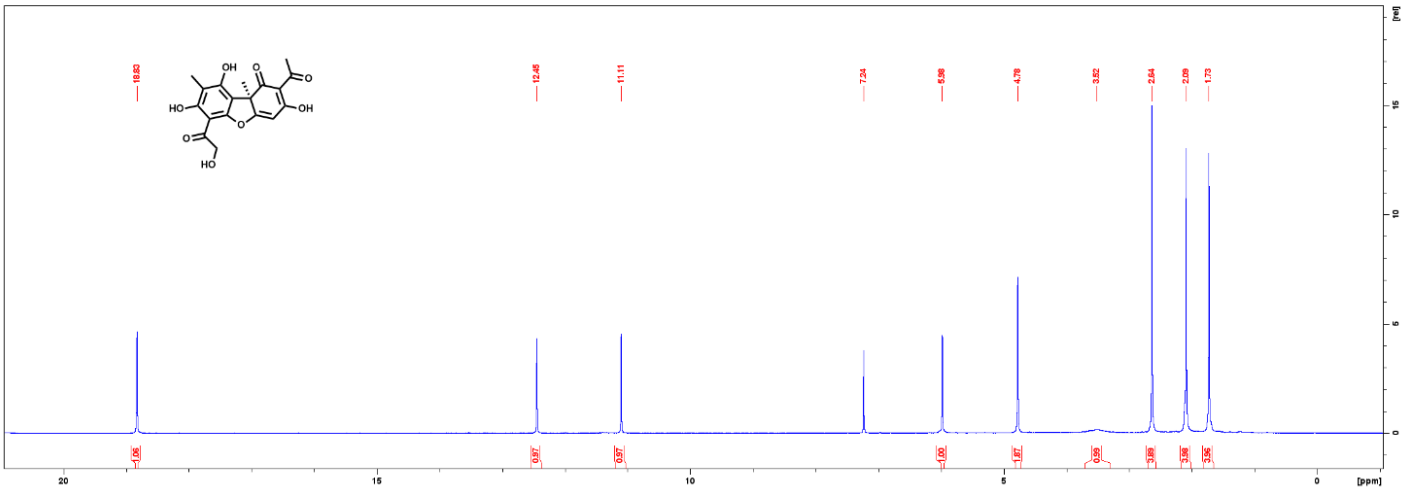

Figure S4. NMR ^{1}H spectrum of **10.**

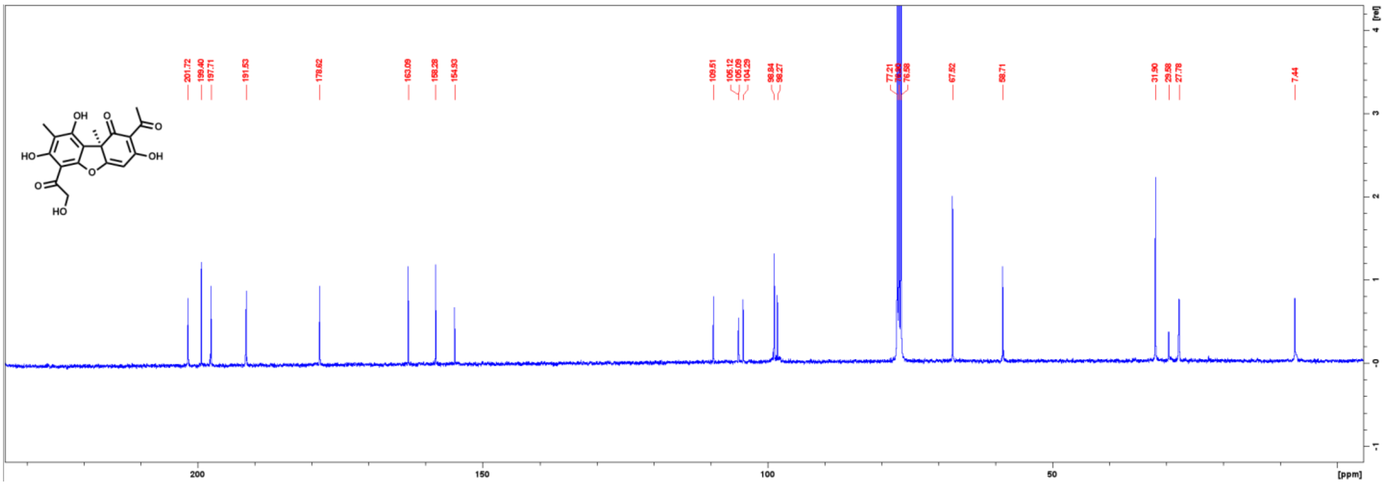

Figure S5. NMR ^{13}C spectrum of 10.

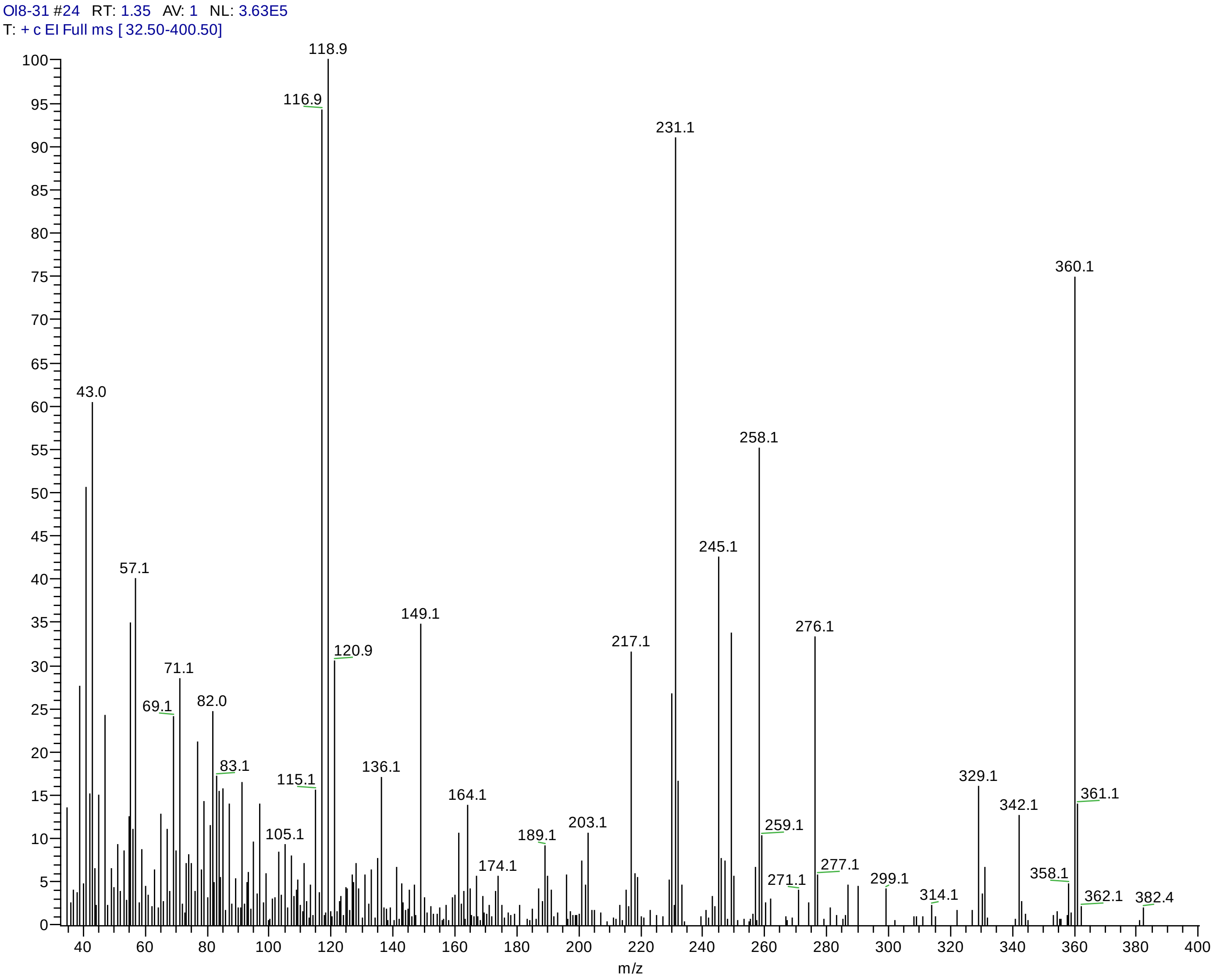

Figure S6. DFS spectrum of **10.**

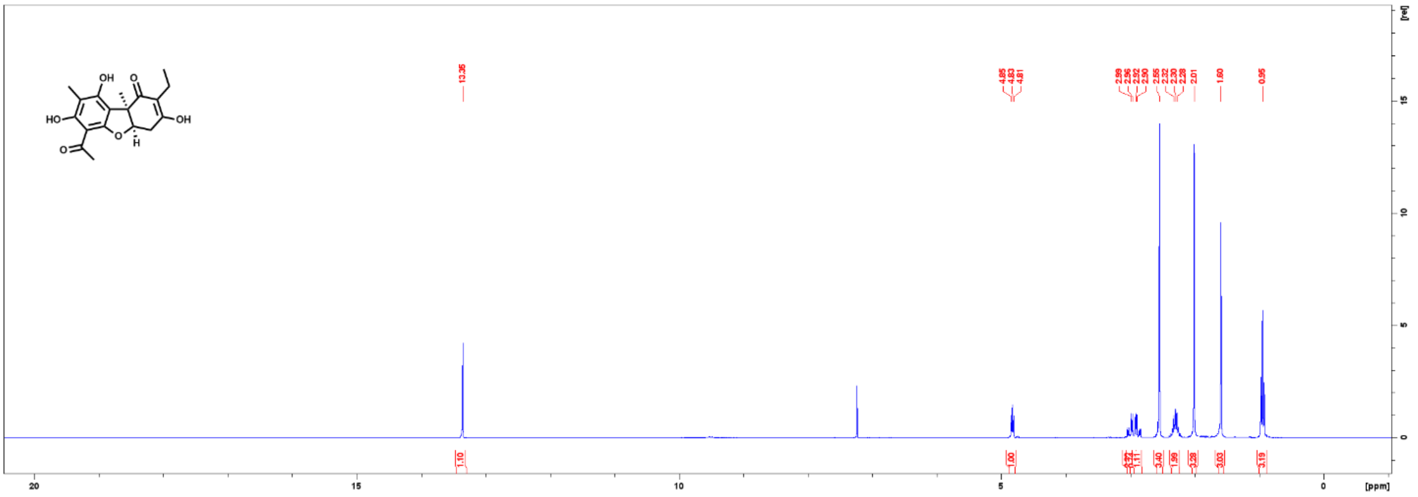

Figure S7. NMR ^{1}H spectrum of **12.**

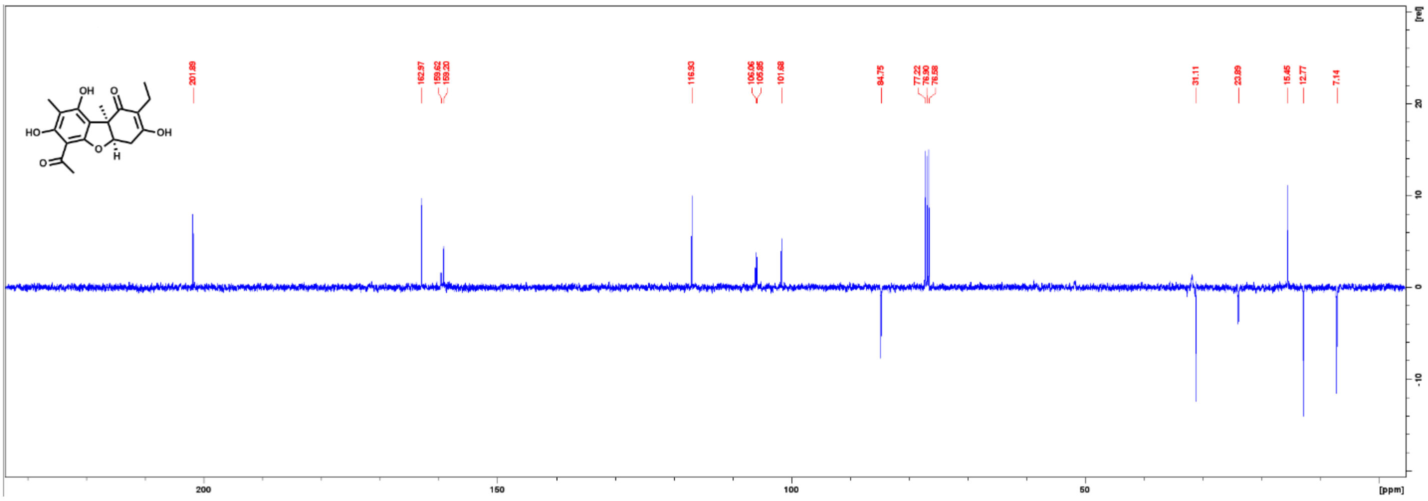

Figure S8. NMR ^{13}C spectrum of 12.

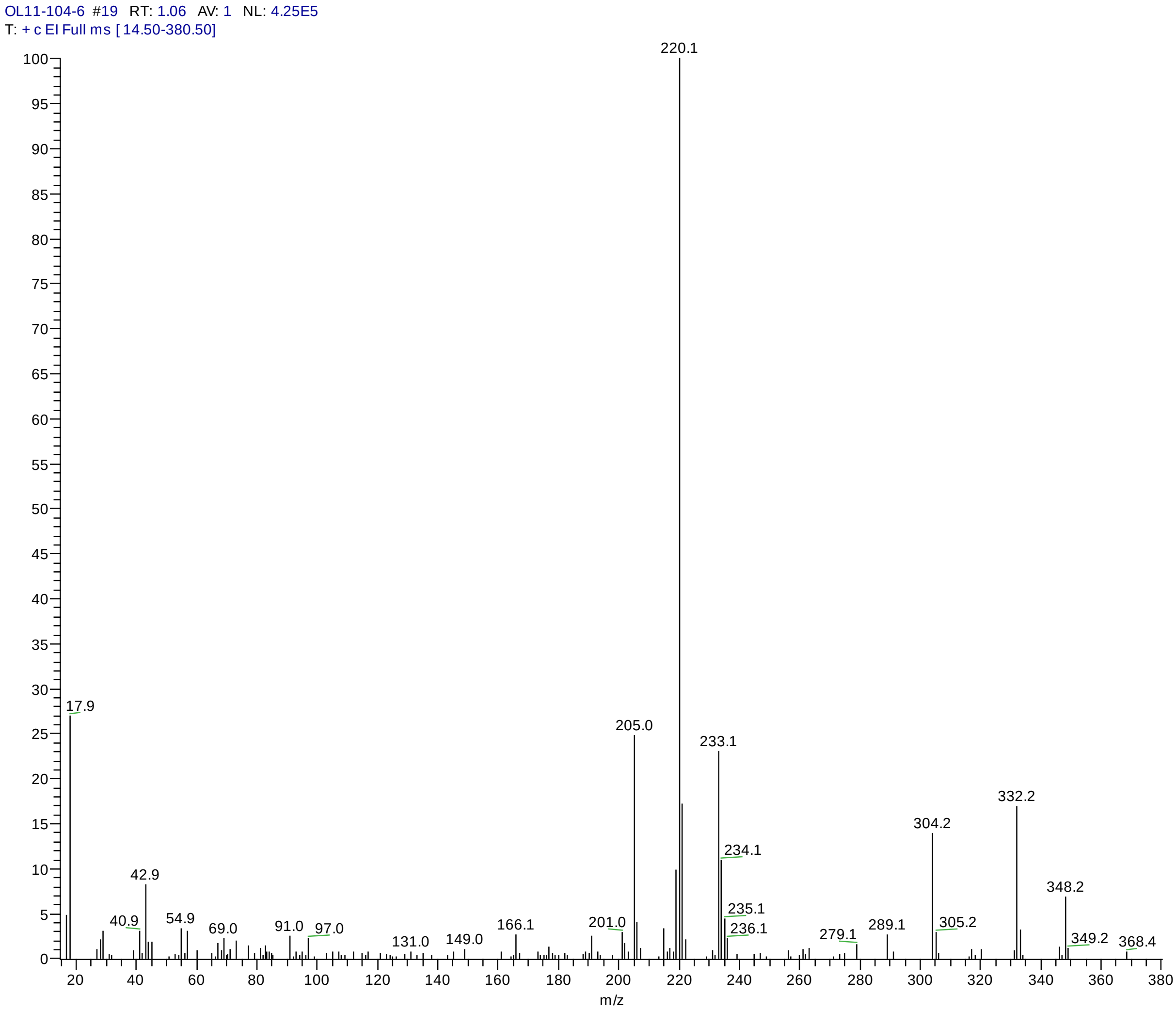

Figure S9. DFS spectrum of 12.

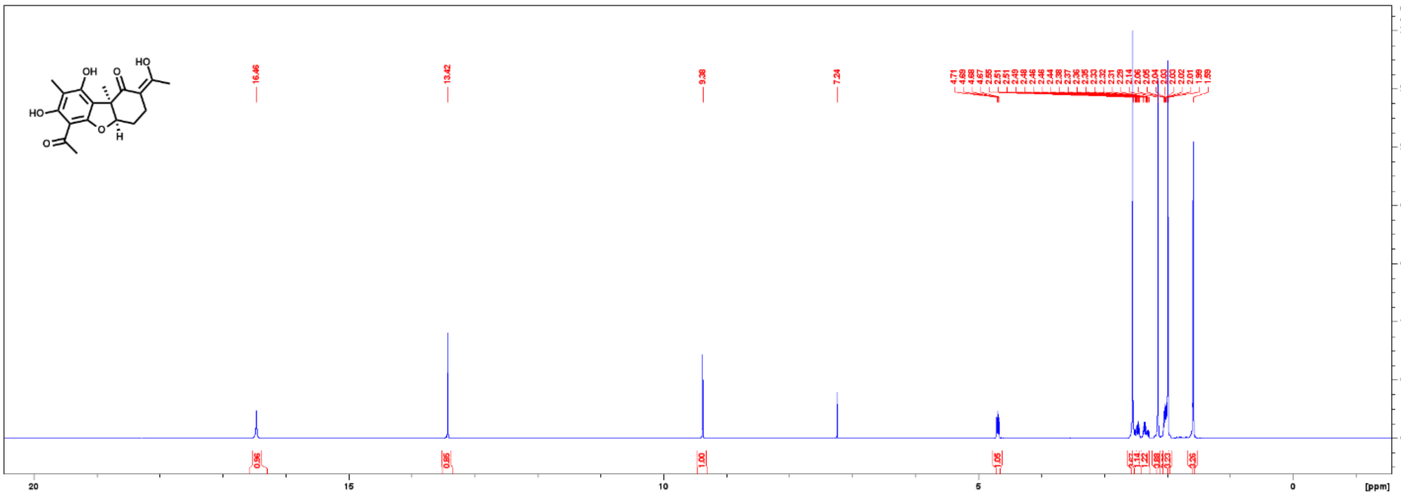

Figure S10. NMR ^{1}H spectrum of 13.

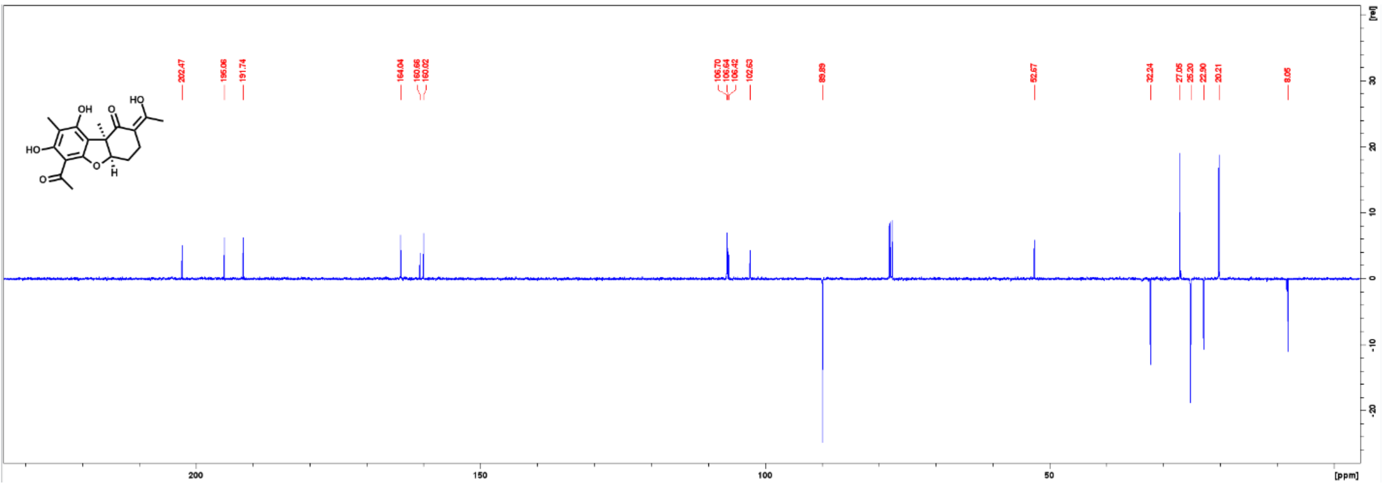

Figure S11. NMR ^{13}C spectrum of 13.

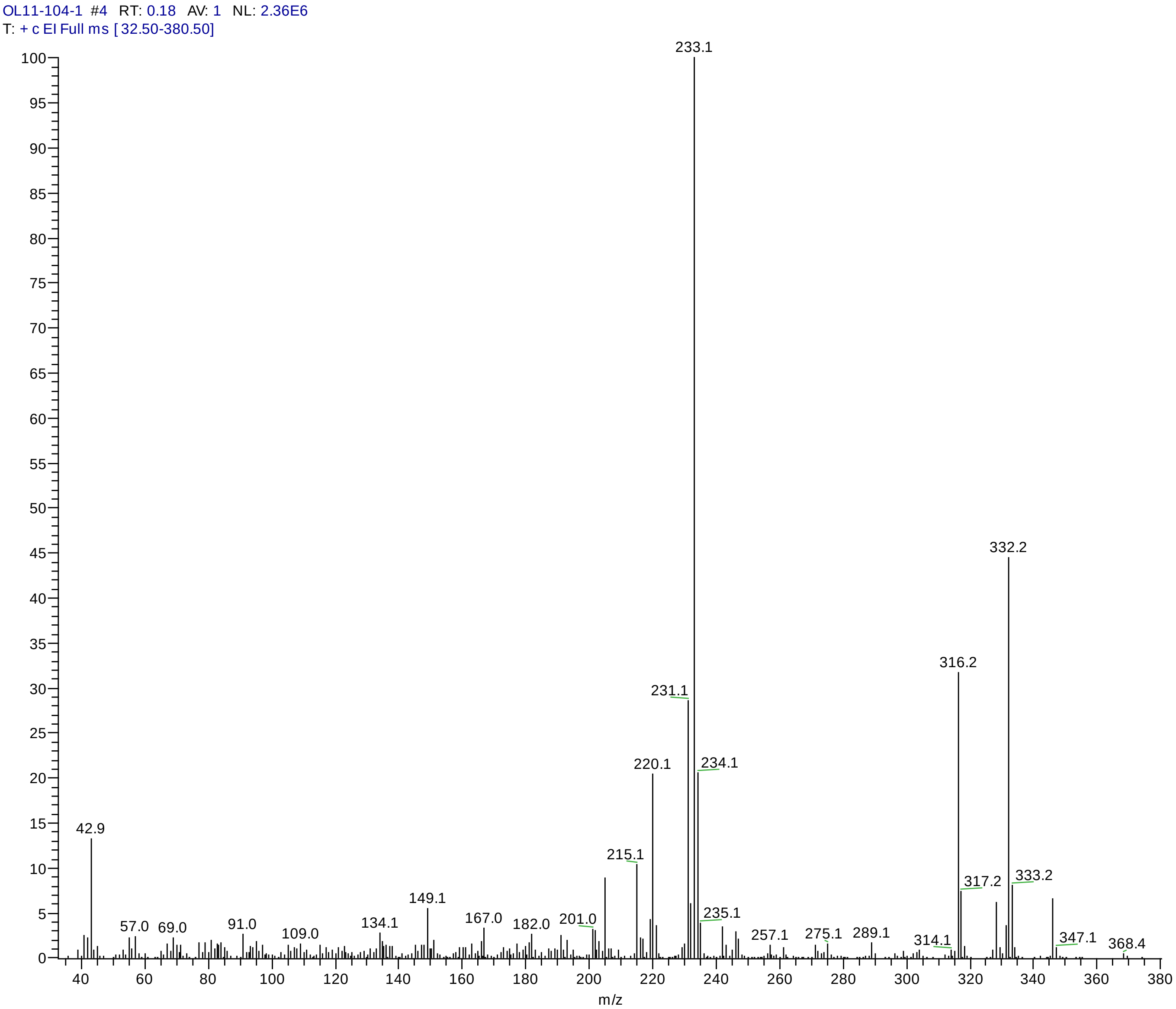

Figure S12. DFS spectrum of 13.

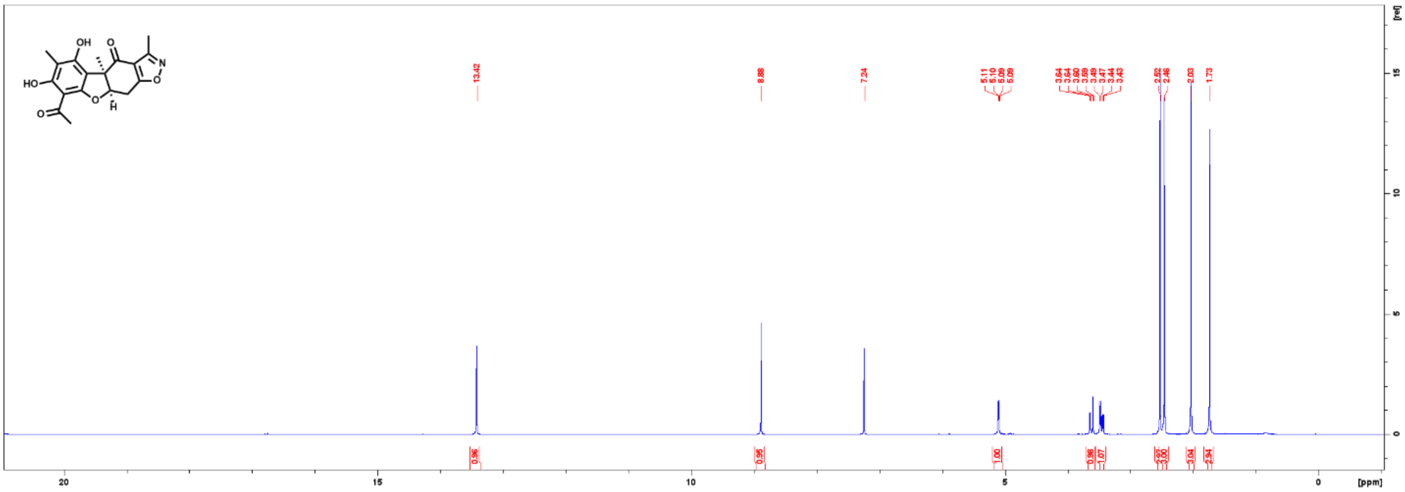

Figure S13. NMR ^{1}H spectrum of 14.

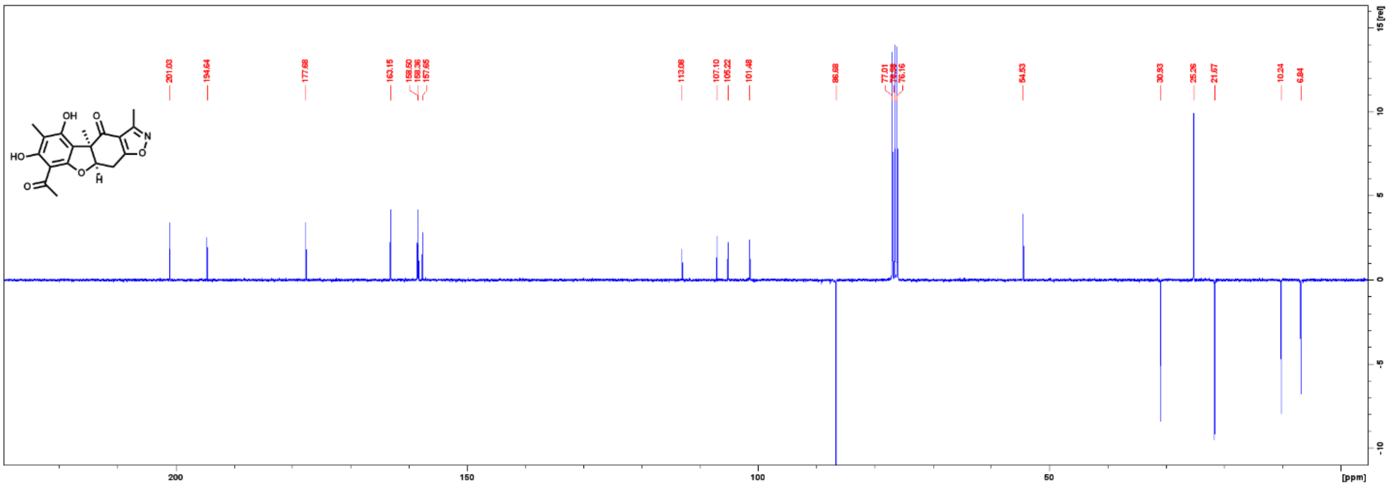

Figure S14. NMR ^{13}C spectrum of 14.

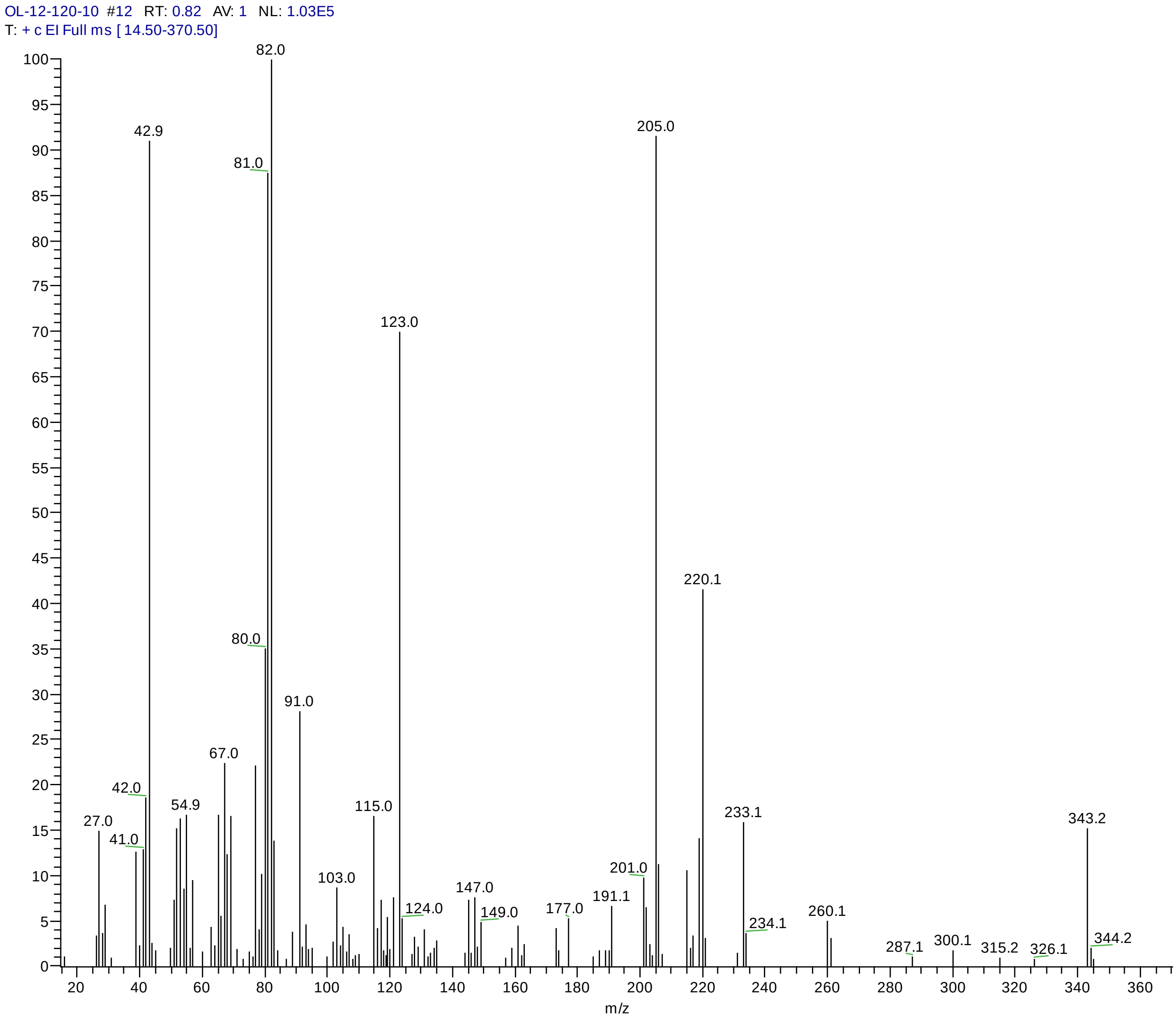

Figure S15. DFS spectrum of 14.

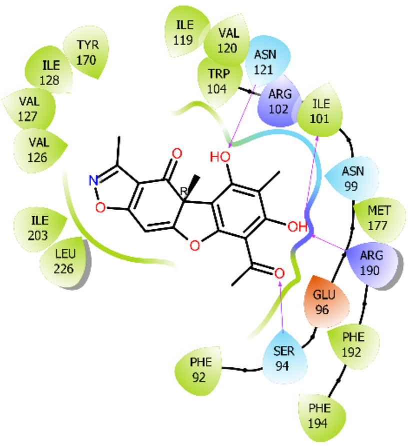

2

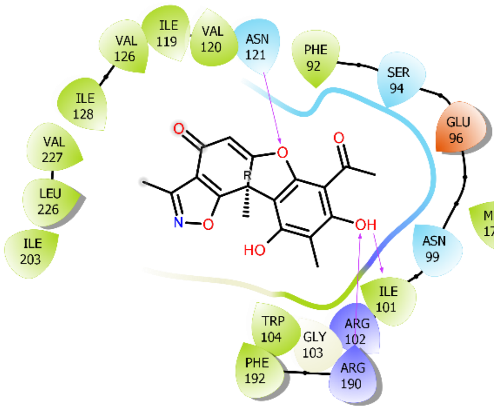

3

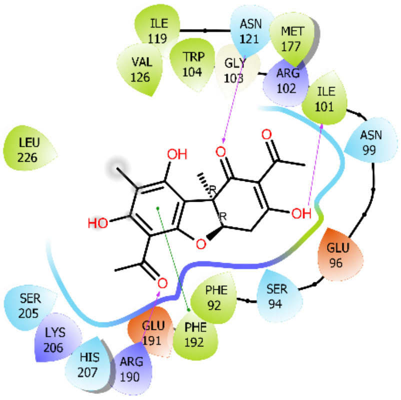

11

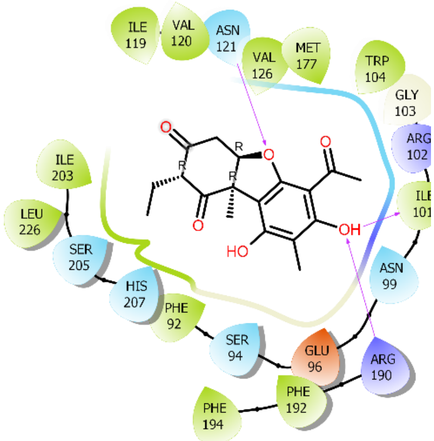

12

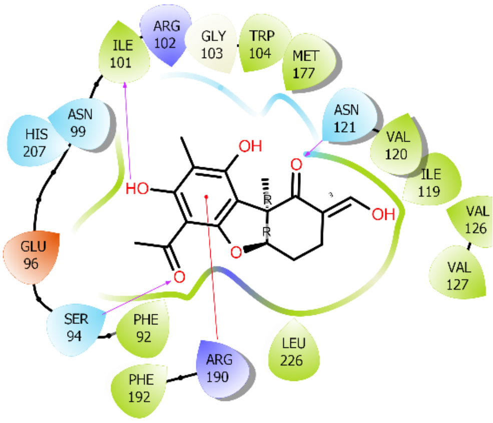

13

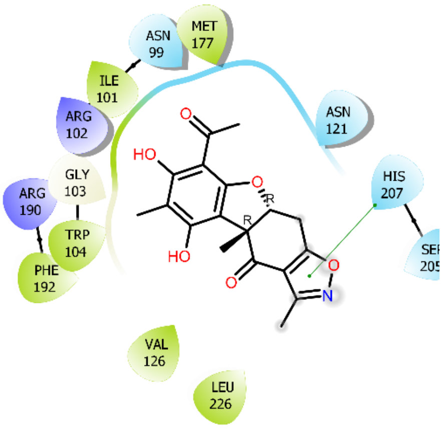

14

Figure S16. Location ligands in the biliverdin binding site.

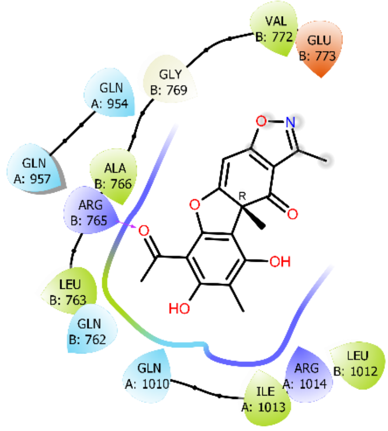

2

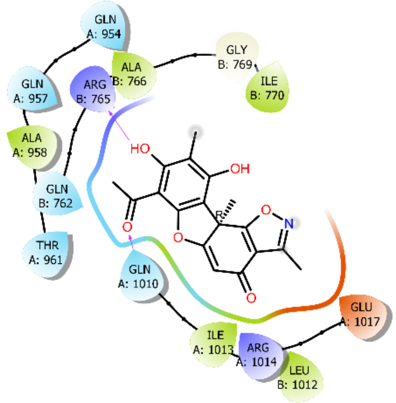

3

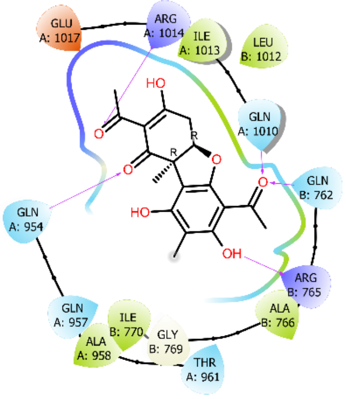

11

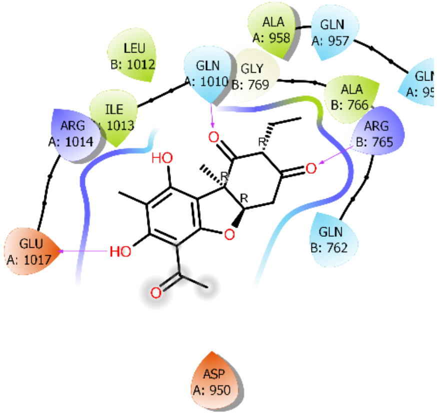

12

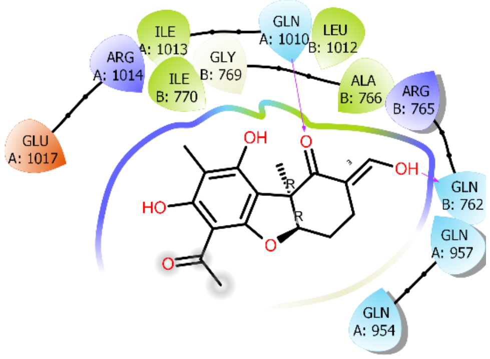

13

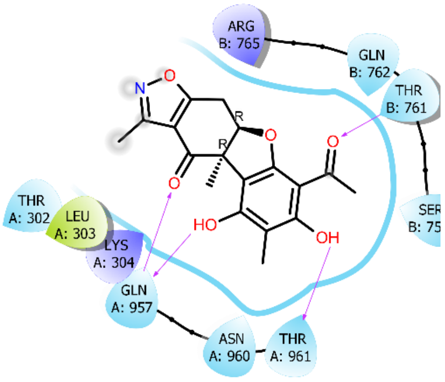

14

Figure S17. Location ligands in the UA-30 and nelfinavir binding site.

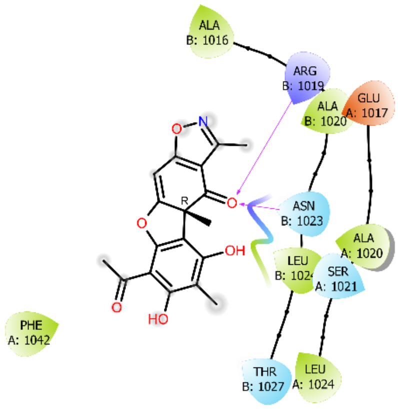

2

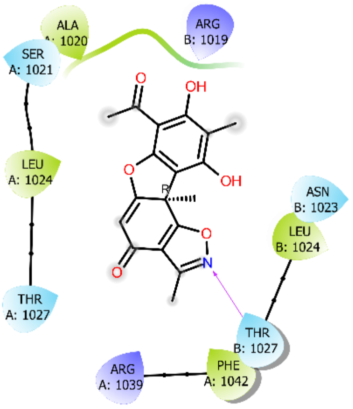

3

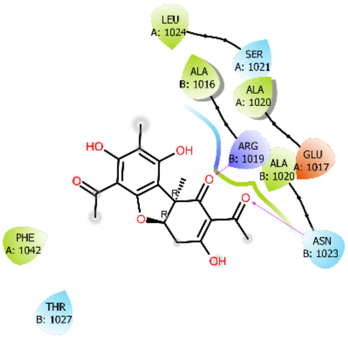

11

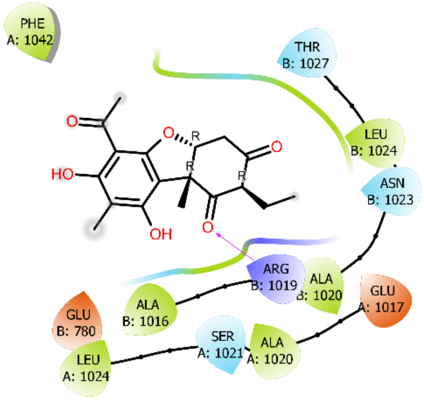

12

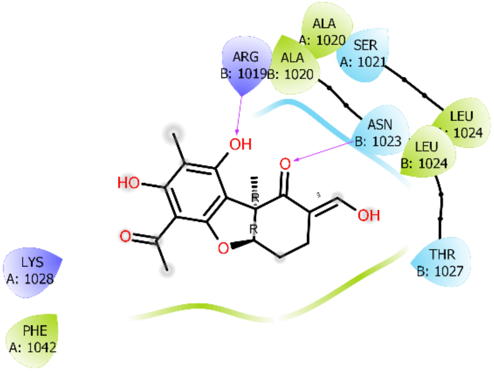

13

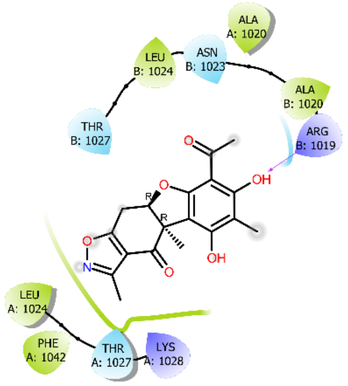

14

Figure S18. Location ligands in the umifenovir binding site.

Table S1. Molecular modeling results.

#	Structure	In Vivo (IC_{50})	In Vitro (Pseudo-type-Based Assay)	Matched Ligand Site	Glide Score	Emodel	IFD Score	H-Bond	Other Interaction	Clash
	BILIVERDIN BINDING SITE			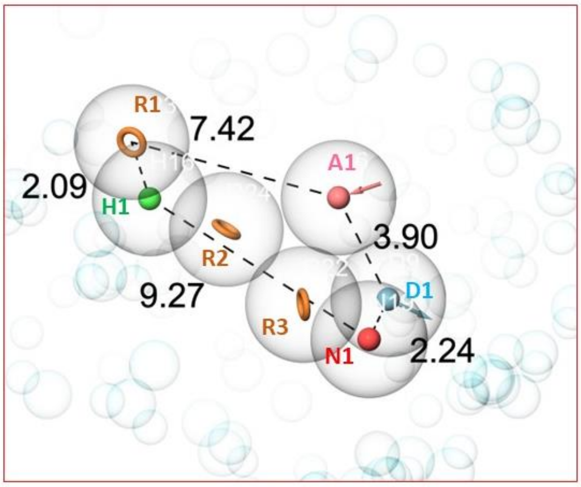						
2	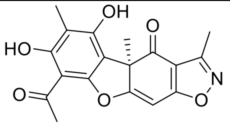 Chemical Formula: $C_{18}H_{15}NO_6$ Molecular Weight: 341,31	n/a	yes	R1; H1; R2; A1	−6.118	−43.497	−614.43	Asn121, Ser94 Arg120, Ile101,	Leu226, Ile203, Trp104 hydro-phobic	Asn121
3	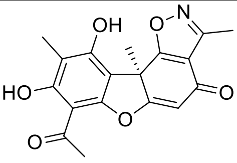 Chemical Formula: $C_{18}H_{15}NO_6$ Molecular Weight: 341,31	9.2	> 25	R1; H1; R2; A1	−8.124	−57.934	−613.90	Asn121, Arg120, Ile101,	Trp104 hydro-phobic	
14	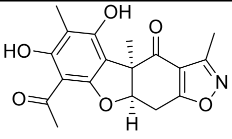 Chemical Formula: $C_{18}H_{17}NO_6$ Molecular Weight: 343,33	n/a	n/a	H1; R2; A1	−5.406	−43.406	−615.87	none	His207 pi-pi stacking; Ile101, Trp104 hydro-phobic	Ile101
11	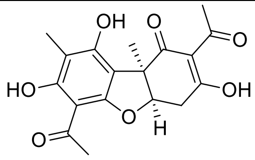 Chemical Formula: $C_{18}H_{18}O_7$ Molecular Weight: 346,33	21.0	22	R1; H1; D1; R3	−7.465	−63.583	−617.77	Asn121, Ile101, Arg190	Phe190 pi-pi stacking	
12	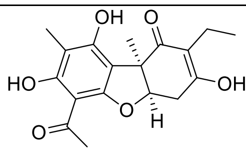 Chemical Formula: $C_{18}H_{20}O_6$ Molecular Weight: 332,35	91.0	>25 (5)	H1; R2; A1	−8.116	−55.76	−616.79	Asn121, Ile101, Arg190	Ile203, Phe92, Trp104 hydro-phobic	

13	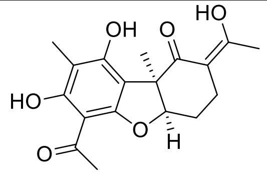<br>Chemical Formula: $C_{18}H_{20}O_6$<br>Molecular Weight: 332,35	6.7	5	H1; R2; A1	−8.908	−70.987	−615.62	Asn121; Ile101; Se94	Arg190 – pi-cation;	
	UA-30 AND NELFINAVIR BINDING SITE			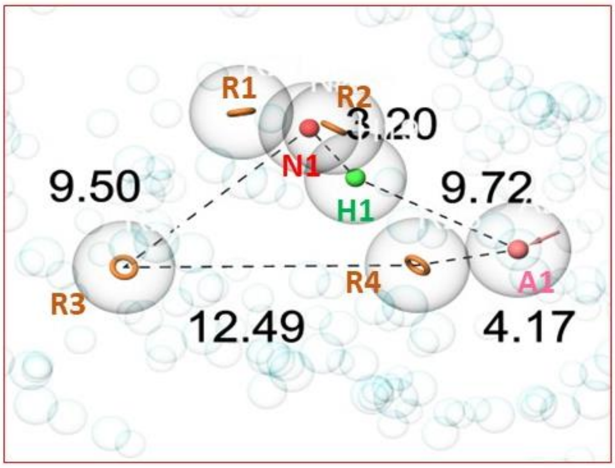 						
2	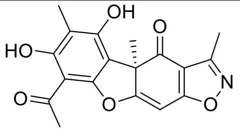<br>Chemical Formula: $C_{18}H_{15}NO_6$<br>Molecular Weight: 341,31	n/a	yes	A1; H1; R4	−6.923	−53.724	−6010.48	B: Arg765	B: Ala766 hydrophobic	
3	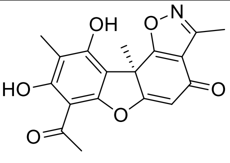<br>Chemical Formula: $C_{18}H_{15}NO_6$<br>Molecular Weight: 341,31	9.2	> 25	A1; H1; R2; R4	−7.513	−63.046	−6011.35	B: Arg765<br>A: Gln1010	A: Leu1013; B: Leu1012.<br>A: Gln954;	B: Arg765
14	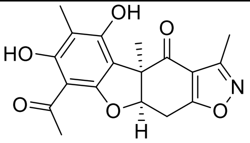<br>Chemical Formula: $C_{18}H_{17}NO_6$<br>Molecular Weight: 343,33	n/a	n/a	R1	−6.192	−48.886	−6011.77	A: Gln957<br>A: Thr961<br>B: Thr761	B: Arg765<br>B: Ser768.	A: Thr302
11	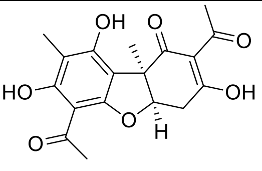<br>Chemical Formula: $C_{18}H_{18}O_7$<br>Molecular Weight: 346,33	21.0	22	R2	−8.806	−66.965	−6014.29	A: Arg765;<br>B: Gln762;<br>A: Gln1010;<br>A: Arg1014;<br>A: Gln954	B: Leu1012 hydrophobic	A: Gln954
12	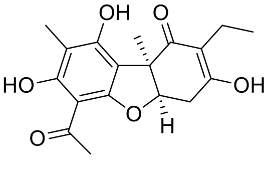<br>Chemical Formula: $C_{18}H_{20}O_6$<br>Molecular Weight: 332,35	91.0	>25 (5)	R2	−6.180	−54.713	−6009.85	A: Gln1010;<br>B: Arg765;<br>A: Glu1017	B: Ala766;<br>B: Leu1012 hydrophobic	

13	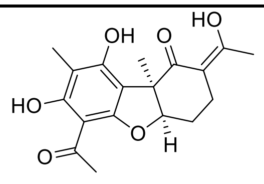<br>Chemical Formula: $C_{18}H_{20}O_6$<br>Molecular Weight: 332,35	6.7	5	R1	−7.263	−59.125	−6010.31	A: Gln1010;	B: Ile770;<br>B: Ala766 hydrophobic	
	UMIFENOVIR BINDING SITE				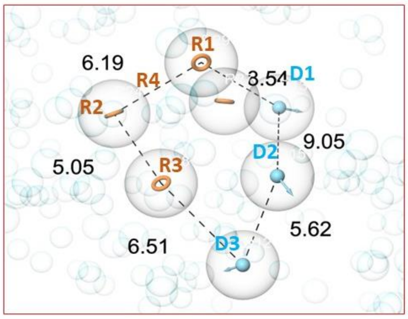					
2	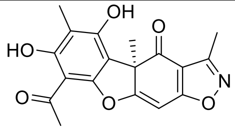<br>Chemical Formula: $C_{18}H_{15}NO_6$<br>Molecular Weight: 341,31	n/a	yes	D3; R3; R1	−4.599	−34.606	−6085.57	B: Arg1019<br>B: Asn1023	B: Arg1019 hydrophobic	
3	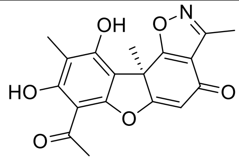<br>Chemical Formula: $C_{18}H_{15}NO_6$<br>Molecular Weight: 341,31	9.2	> 25	R3	−4.362	−36.163	−6085.57	B: Thr1027		B: Arg1019
14	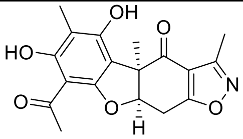<br>Chemical Formula: $C_{18}H_{17}NO_6$<br>Molecular Weight: 343,33	n/a	n/a	D3; R3; R2	−6.527	−6.527	−6180.97	B: Arg1019	B: Ala1020; A: Leu1024; A: Phe1042 hydrophobic	B: Thr1027
11	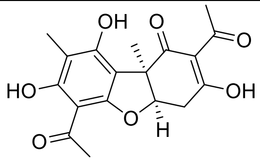<br>Chemical Formula: $C_{18}H_{18}O_7$<br>Molecular Weight: 346,33	21.0	22	D3; R3; D2	−5.151	−43.303	−6179.08	B: Arg1019<br>B: Asn1023	B: Ala1020; B: Ala1016 hydrophobic;	B: Arg1019; A: Ala1020; A: Leu1024
12	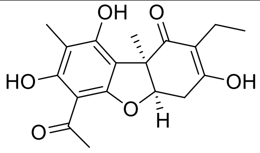<br>Chemical Formula: $C_{18}H_{20}O_6$<br>Molecular Weight: 332,35	91.0	>25 (5)	R3	−4.32	−36.64	−6081.82	B: Arg1019	B: Leu 1024; A: Phe1042; hydrophobic	B: Arg1019

13	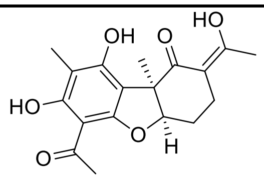 Chemical Formula: $C_{18}H_{20}O_6$ Molecular Weight: 332,35	6.7	5	D3; R3	−4.288	−35.676	−6081.82	B: Arg1019 B: Asn1023	B: Leu 1024; B: Ala1020 hydrophobic
